# CircIQGAP1-CARM1 axis promotes renal cell carcinoma progression through glycolytic reprogramming

**DOI:** 10.1038/s41419-026-08661-w

**Published:** 2026-03-27

**Authors:** Ruyue Jia, Benkui Zou, Yiran Liang, Qingkun Chen, Tao Chen, Xiangzhi Li, Chao Zhang, Fajun Pei, Xuehua Zhu, Jiasheng Bian, Renbo Guo

**Affiliations:** 1 Department of Urology, Shandong Cancer Hospital and Institute, Shandong First Medical University and Shandong Academy of Medical Sciences, Jinan, China; 2 Shandong Provincial Key Laboratory of Precision Oncology, Cancer Research Center, Shandong Cancer Hospital and Institute, Jinan, Shandong Province China; 3 Department of Breast Surgery, General Surgery, Qilu Hospital of Shandong University, Jinan, China; 4 Shandong Provincial Key Laboratory of Animal Cell and Developmental Biology, School of Life Sciences, Shandong University, Qingdao, China

**Keywords:** Renal cell carcinoma, RNA splicing

## Abstract

Renal cell carcinoma (RCC) relies heavily on aerobic glycolysis for rapid proliferation and metastasis; however, the role of circular RNAs (circRNAs) in this process remains unclear. This study identified circIQGAP1 as a key regulator; its expression was upregulated under glucose deficiency, with U2AF2 promoting its biosynthesis. Functional assays showed that circIQGAP1 enhances RCC cell proliferation, motility, invasion, and glycolytic flux. Mechanistically, circIQGAP1 binds to CARM1, inhibiting its K48-linked ubiquitination and prolonging its half-life. Elevated CARM1 then catalyses COL5A1 promoter dimethylation, driving COL5A1 transcription. Rescue experiments confirmed that both CARM1 and COL5A1 were essential for circIQGAP1-mediated metabolic reprogramming and malignant phenotypes. Clinically, circIQGAP1 is overexpressed in RCC tissues and is correlated with poor outcomes. These findings revealed that circIQGAP1 promotes glycolysis-dependent RCC progression by stabilizing CARM1 to activate COL5A1, highlighting that this regulatory axis may provide an innovative strategy for RCC treatment.

## Introduction

Renal cell carcinoma (RCC) is a common urological malignancy with rising global incidence and mortality. According to the 2020 Global Cancer Statistics, 431,288 new cases of kidney cancer and 179,368 related deaths have been reported worldwide [1]. Clear cell RCC (ccRCC) accounts for more than 80%, poses significant clinical challenges owing to its high invasiveness, early metastatic tendency, and molecular heterogeneity [2]. Despite therapeutic advances, recurrence in locally advanced disease and drug resistance in metastatic stages remain major limitations [3, 4]. Notably, 37–61% of RCC cases are incidentally discovered, and 11% of patients are diagnosed at stage IV underscore the critical need for early biomarkers and a deeper understanding of molecular pathogenesis to overcome treatment resistance [5].

Previous studies have revealed that cancer cells predominantly rely on aerobic glycolysis rather than oxidative phosphorylation to sustain energy and metabolic demands, a phenomenon known as the Warburg effect [6, 7]. First described by Otto Warburg in 1924, metabolic reprogramming involves rapid glucose breakdown into lactate, even under oxygen-sufficient conditions, to supply ATP, NADH, and precursors for nucleotide and lipid biosynthesis [8]. Beyond providing ATP and precursors, this metabolic reprogramming in RCC acidifies the tumor microenvironment, modulates epigenetics, and suppresses immune cell function, thereby driving malignant progression [9–11]. Elucidating the regulatory mechanisms of aerobic glycolysis in RCC is crucial for identifying novel therapeutic targets.

Circular RNAs (circRNAs), characterized by covalently closed structures conferring high stability, are emerging as key regulators in cancer [12]. They function by interacting with RNA-binding proteins (RBPs), absorbing miRNAs, or encoding functional peptides, thereby regulating tumor cell proliferation, apoptosis, invasion [13–15], and aerobic glycolysis [16–18]. Dysregulated circRNAs have been linked to disease progression and poor prognosis [19–21]. However, the mechanisms by which circRNAs modulate aerobic glycolysis to drive RCC progression remain largely unexplored. Here, we identified circIQGAP1 as a cancer-related candidate through transcriptome profiling of RCC cells under low glucose stress combined with public dataset analysis. CircIQGAP1 was upregulated by U2AF2 and promoted malignant progression and aerobic glycolysis in RCC. Mechanistically, circIQGAP1 binds and stabilizes the arginine methyltransferase CARM1, leading to transcriptional upregulation of the glycolysis-related gene COL5A1. Our findings not only broadened the current understanding of metabolic reprogramming in RCC but also highlighted novel molecular targets for therapeutic intervention.

## Materials and methods

### Cell culture and transfection

Human RCC cell lines 786-O and 769-P were maintained in RPMI-1640 medium supplemented with 10% fetal bovine serum (FBS) at 37 °C in a humidified incubator with 5% CO₂. Cell lines were routinely tested for mycoplasma contamination and confirmed negative. Transfections were performed using Lipofectamine 2000 with pLCDH-ciR, pcDNA3.1 plasmids, or siRNAs targeting the following sequences: U2AF2, 5′-GTGAGTACGTGGACATCAA-3′; CARM1, 5′-GTACACGGTGAACTTCTTA-3′; and COL5A1, 5′-GGGATTCCTTCAAGGTTTA-3′. Transfection efficiency was validated by quantitative real-time reverse transcription polymerase chain reaction (qRT-PCR).

### qRT-PCR and Western blotting

Total RNA and proteins were extracted from the transfected and control cells. For RT-qPCR, RNA was reverse-transcribed into cDNA and amplified with target-specific primers, with β-actin as the internal control. For western blotting, protein lysates were resolved by 10% sodium dodecyl sulfate-polyacrylamide gel electrophoresis, electrotransferred to polyvinylidene difluoride membranes, and incubated with target-specific antibodies, with glyceraldehyde-3-phosphate dehydrogenase (GAPDH) as an internal reference. Antibodies against CARM1 (55246-1-AP), U2AF2 (15624-1-AP), COL5A1 (67604-1-Ig), actin (66009-1-Ig), GAPDH (60004-1-Ig), ubiquitin (Ub, 10201-2-AP) and Ki67 (27309-1-AP) were purchased from Proteintech (Wuhan, China). Anti-Flag (DYKDDDDK) tag (14793) was purchased from Cell Signaling Technology (MA, USA). Anti-K48 (AB140601) and anti-K63 (AB179434) were purchased from Abcam (Cambridge, UK).

### CircRNA circularization validation

To confirm the circular nature of the candidate RNA, total RNA extracted from RCC cells was treated with RNase R for 30 min at 37 °C and then inactivated at 70 °C. In parallel, RNA samples were treated with Random 6M primers and oligo dT primers to synthesize cDNA. The resulting cDNA samples were used as templates for PCR amplification and then analyzed.

### Actinomycin D treatment

To assess RNA stability, RCC cells were treated with actinomycin D to inhibit RNA transcription. Total RNA was collected 0, 4, 8, 12, and 24 h after treatment. The residual RNA levels of the circular and linear transcripts were quantified using qRT-PCR.

### Cytosolic/nuclear fraction assay

After cell lysis, cytoplasmic and nuclear fractions were separated using a kit. Subsequently, RNA was extracted from each fraction, and the expression levels of circRNAs were assessed to determine their subcellular localization.

### Apoptosis analysis by flow cytometry

Cells were harvested, washed with phosphate-buffered saline, and stained with Annexin V-FITC and propidium iodide (PI), according to the manufacturer’s protocol. After incubation in the dark, cells were analyzed using a flow cytometer. Early apoptotic cells (Annexin V+/PI−) and late apoptotic cells (Annexin V+/PI+) were quantified, and the percentage of total apoptotic cells was calculated.

### Cell proliferation assays

The 3-(4,5-dimethylthiazol-2-yl)-2,5-diphenyltetrazolium bromide (MTT) assay was used to measure cell proliferation. Transfected cells were seeded in 96-well plates, incubated for 5 days, and treated with the MTT reagent. The absorbance at 570 nm was measured after solubilization of dimethyl sulfoxide. For the colony formation assay, the transfected cells were plated in 6-well plates and cultured for 14 days. colonies were fixed, stained, and counted.

### Migration and invasion assays

For the wound-healing assay, transfected cells were seeded in 6-well plates and grown to 90% confluence. A sterile pipette tip was used to create a linear scratch, and wound closure was monitored at 0 and 24 h. For the Transwell invasion assay, transfected cells in serum-free medium were added to Matrigel-coated Transwell chambers with FBS as a chemoattractant. After 24 h, the invading cells were fixed, stained with crystal violet, photographed, and quantified.

### Lactate and ATP production

Lactate in cell culture supernatants was quantified using a lactate assay kit. For ATP measurements, intracellular ATP levels were assessed using an ATP assay kit.

### Glycolytic stress assay

Glycolytic function was assessed using a Seahorse XF Analyzer. Cells were sequentially treated with glucose to initiate glycolysis, oligomycin to inhibit mitochondrial ATP production, and 2-deoxyglucose (2-DG) to inhibit glycolysis and to measure non-glycolytic acidification. The extracellular acidification rate (ECAR) was monitored and key parameters, including basal glycolysis, glycolytic capacity, and glycolytic reserve, were calculated.

### RNA immunoprecipitation (RIP) and co-immunoprecipitation (Co-IP)

RCC cell lysates were incubated with specific antibodies and Protein A/G magnetic beads. After extensive washing to remove non-specific binding, immune complexes were collected. For the RIP assays, RNA was extracted from the precipitates and analyzed by qRT-PCR to identify RNA molecules bound to the target protein. For co-IP assays, proteins were eluted, and the target protein and its interacting proteins were detected by western blotting.

### Patients and samples

Renal cancer tissues were collected from patients with RCC who underwent surgery at the Shandong Cancer Hospital and Institute. Written informed consent was obtained from all participants. This study was approved by the Ethics Review Committee of Shandong Cancer Hospital and Institute (Approval no. SDTHEC2023001023) and conducted in strict accordance with the ethical principles of the Declaration of Helsinki.

### Mouse xenograft model

Four-week-old female BALB/c nude mice were obtained from Vital River Laboratory Animal Technology Co., Ltd (Beijing, China). Animals were randomly allocated into groups (*n* = 5) and subcutaneously inoculated with RCC cells stably overexpressing circIQGAP1 or control vector pLCDH-ciR. Mice were excluded from analysis if tumor engraftment failed or if they developed ulceration at the injection site. These exclusion criteria were pre-established in the animal study protocol. Tumor size was measured every five days, and the volume was calculated according to the formula (length × width²)/2. After 30 days, subcutaneous tumors were collected, weighed, and processed for histological (HE) staining and immunohistochemical (IHC). All animal procedures were approved by the Ethics Review Committee of Shandong Cancer Hospital and Institute (Approval no. SDTHEC2023001023) and performed in compliance with institutional ethical guidelines.

### Statistical analysis

All statistical analyzes were conducted using GraphPad Prism 8.0 software. At least three independent biological replicates were performed for each experiment, and the data are presented as mean ± standard deviation (SD). Differences between two groups were evaluated using Student’s *t*-test, whereas comparisons among multiple groups were performed via ANOVA. Kaplan–Meier survival curves were used to assess differences in patient prognosis with the log-rank test. Additionally, the data meets the assumptions of the test, including the variations and variances within each group. All *p*-values were calculated using two-tailed tests, and statistical significance was defined as *p* < 0.05.

## Results

### CircIQGAP1 is identified as a glucose stress-responsive circRNA in RCC

**Fig. 1:**
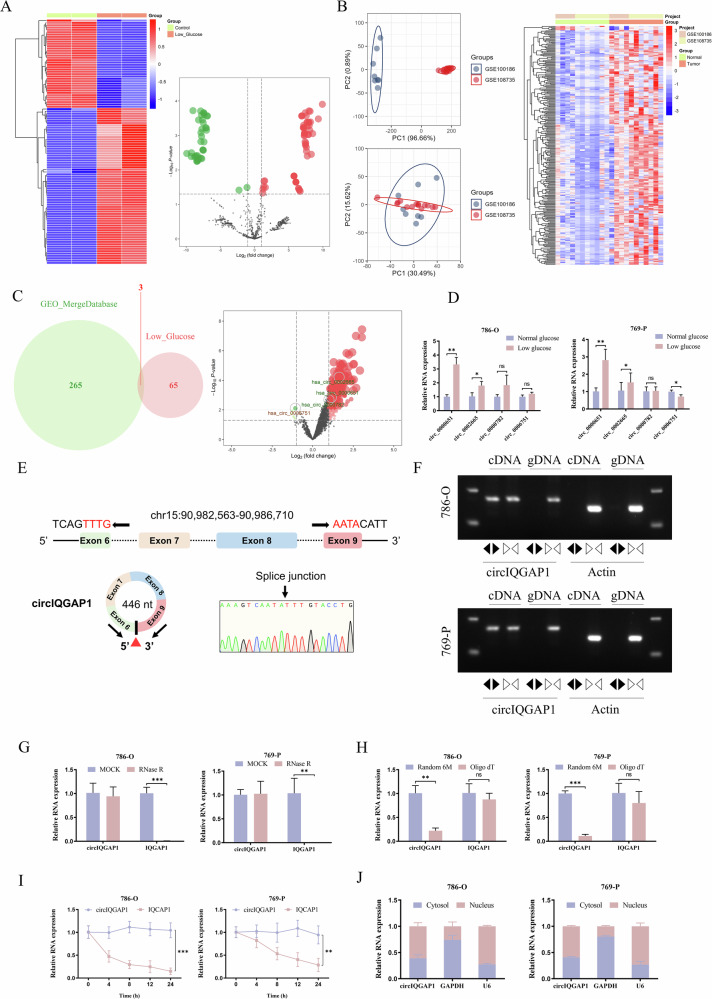
CircIQGAP1 is identified as a glucose stress-responsive circRNA in RCC cells. **A** Heatmap and volcano plot of differentially expressed circRNAs from RNA-seq of RCC cells cultured in low vs. normal glucose. **B** Principal component analysis (PCA) and heatmap of differentially expressed genes in GSE100186 and GSE108735 public datasets. **C** Venn diagram and volcano plot showing overlap between low glucose stress-responsive circRNAs and oncogenes identified from GEO public datasets. **D** qRT-PCR validation of expression changes for four candidate circRNAs in RCC cells following low glucose treatment. **E** Genomic location and circularization schematic of circIQGAP1, along with Sanger sequencing of the back-splicing junction. **F** Divergent and convergent primer PCR on cDNA and gDNA to confirm circIQGAP1 circularity. **G** RNase R resistance assay comparing circIQGAP1 and linear IQGAP1 mRNA levels in RCC cells. **H** Reverse transcription with Oligo (dT) and Random 6M primers to detect the absence of a 3’ poly(A) tail in circIQGAP1. **I** Half-life comparison of circIQGAP1 and linear IQGAP1 mRNA after actinomycin D treatment. **J** Subcellular fractionation assay to assess intracellular localization of circIQGAP1 in RCC cells, U6 and GAPDH serve as nuclear and cytoplasmic controls, respectively. Data are shown as the mean ± SD of at least three independent experiments, and the significant level was identified by ns, no significance, ^*^P < 0.05, ^**^P < 0.01, and ^***^P < 0.001.

To investigate glucose availability’s effect on RCC metabolic reprogramming, 786-O and 769-P cells were cultured under low- or normal-glucose conditions. The low-glucose group showed significantly higher extracellular lactate secretion and intracellular ATP levels, indicating increased aerobic glycolysis to maintain energy homeostasis under nutrient stress (Fig. S1A, B). To explore circRNAs’ role in metabolic stress adaptation, circRNA sequencing on RCC cells under both glucose conditions identified differentially expressed circRNAs (Fig. 1A). Public datasets GSE100186 and GSE108735 were analyzed to screen oncogenic differentially expressed genes (Fig. 1B). Integrated analysis identified three candidate circRNAs that were upregulated under low-glucose conditions and concurrently overexpressed in RCC tissues (Fig. 1C). qRT-PCR in low-glucose-treated cells confirmed circIQGAP1 (has_circ_0000651) had the highest expression (Fig. 1D), selected for further study. Interactome and Ensembl databases showed that circIQGAP1, located at chr15:90,982,563-90,986,710, is formed by back-splicing of IQGAP1 exons 6-9 (446 nt). Sanger sequencing identified its back-splicing junction, and secondary structure was predicted (Figs. 1E and S2). qRT-PCR showed divergent primers amplified circIQGAP1 from cDNA but not genomic DNA (Fig. 1F). RNase R treatment obviously degraded linear IQGAP1 mRNA while circIQGAP1 remains stabilized (Fig. 1G). Lacking a poly(A) tail, it was amplified by Random 6 M but not Oligo dT primers (Fig. 1H). Actinomycin D assays showed its longer half-life than linear mRNA (Fig. 1I). Subcellular fractionation revealed nuclear enrichment, suggesting transcriptional/epigenetic regulatory potential (Fig. 1J).

### CircIQGAP1 drives RCC progression through aerobic glycolysis activation

**Fig. 2:**
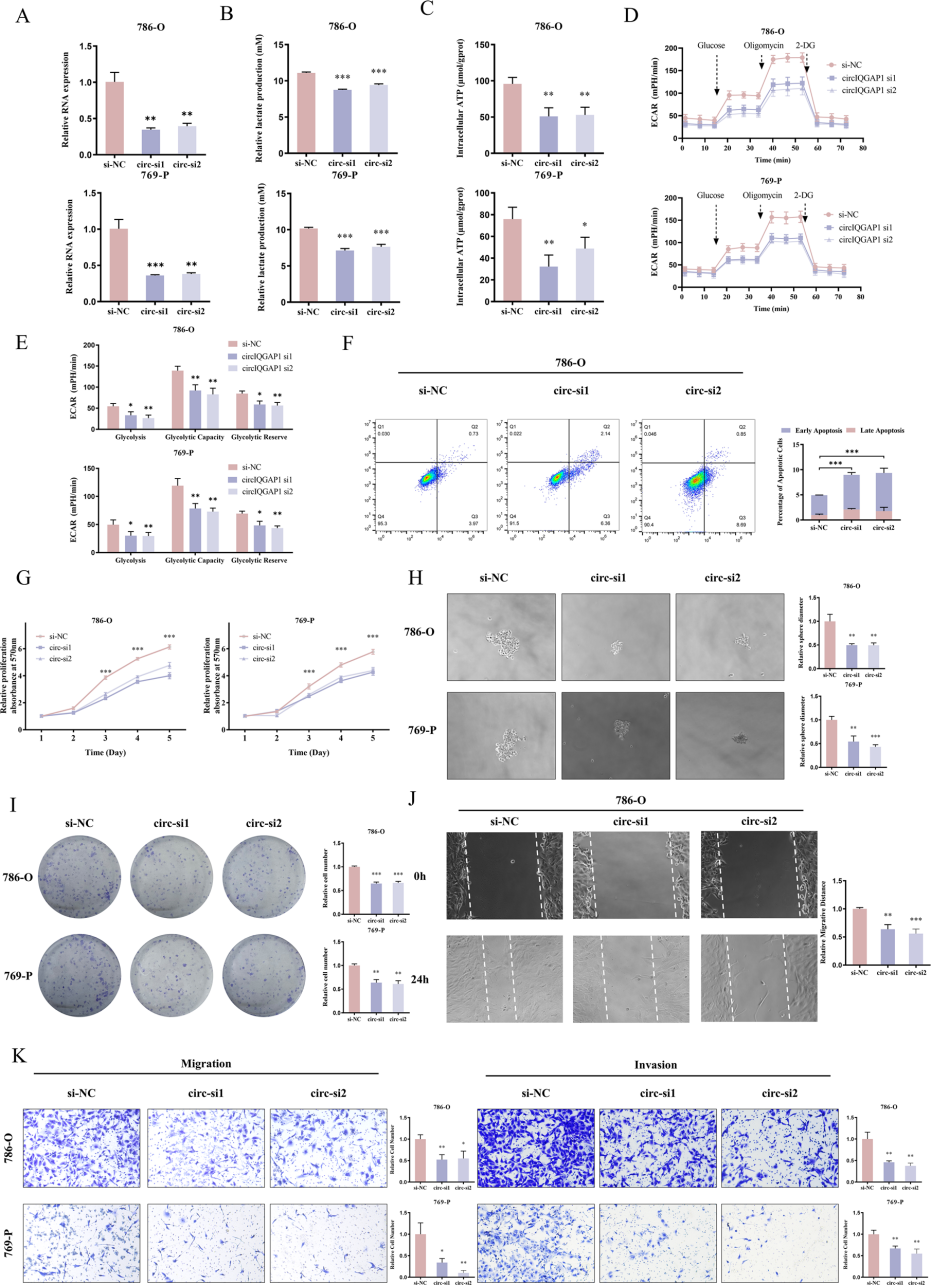
Knockdown of circIQGAP1 suppresses glycolysis and oncogenic behaviors in RCC cells. **A** Specific detection of circIQGAP1 mRNA after knockdown in RCC cells. Lactate production (**B**) and intracellular ATP levels (**C**) in RCC cells following circIQGAP1 knockdown. Extracellular acidification rate (ECAR) (**D**) of RCC cells after circIQGAP1 depletion, and the glycolytic parameters (basal glycolysis, glycolytic capacity, and glycolytic reserve) (**E**) were evaluated. **F** Flow cytometry analysis of apoptosis rates in RCC cells following circIQGAP1 knockdown. MTT assay (**G**), tumor sphere formation (**H**), and colony formation (**I**) to evaluate proliferation and tumorigenic potential following circIQGAP1 knockdown. Wound-healing (**J**) and Transwell (**K**) assays to analyze migratory and invasive capacities after circIQGAP1 depletion. Data are shown as the mean ± SD of at least three independent experiments and the significant level was identified by ^*^P < 0.05, ^**^P < 0.01, and ^***^P < 0.001.

To clarify circIQGAP1’s role in aerobic glycolysis regulation, two siRNAs targeting its back-splice junction were designed, which effectively reduced circIQGAP1 expression without affecting linear IQGAP1 mRNA (Figs. 2A and S3A, B). CircIQGAP1 knockdown significantly decreased lactate production, ATP levels (Fig. 2B, C). Moreover, ECAR measurements showed that glycolytic rate, glycolytic capacity, and glycolytic reserve were significantly lower in circIQGAP1-silenced cells than in control cells (Fig. 2D, E). Conversely, its overexpression enhanced glycolytic activity (Fig. S4A–E).

Next, we explored the biological function of circIQGAP1 in RCC. Flow cytometry showed circIQGAP1 knockdown significantly increased RCC cell apoptosis (*p* < 0.001, Fig. 2F). Functional assays revealed it inhibited cell proliferation, tumorigenic potential (Fig. 2G–I). Additionally, wound healing and Transwell assays confirmed that circIQGAP1 knockdown markedly suppressed the migratory and invasive capabilities of RCC cells (Fig. 2J, K). In contrast, circIQGAP1 overexpression enhanced cell proliferation, migration, and invasion (Fig. S4F–I).

**Fig. 3:**
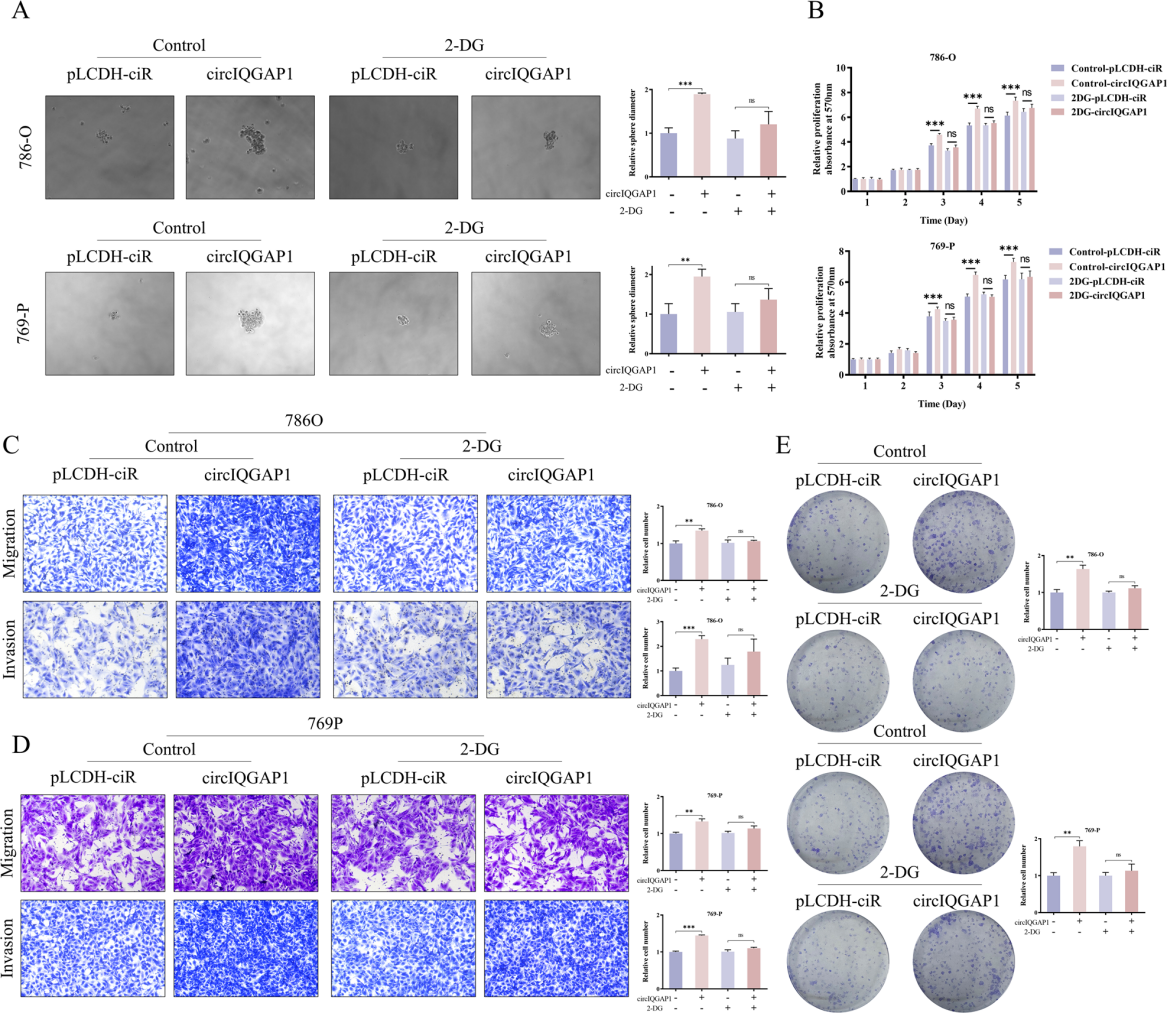
Glycolysis inhibition reverses circIQGAP1-driven malignant phenotypes in RCC. Tumor spheroid formation (**A**) and MTT assays (**B**) assessing the impact of 2-DG on spheroid growth and proliferation in circIQGAP1-overexpressing cells. Transwell assays evaluating migration (**C**) and invasion (**D**) following glycolysis blockade in RCC cells overexpressing circIQGAP1. **E** Colony formation assay measuring the effect of 2-DG on clonogenic capacity of circIQGAP1-overexpressing cells. Data are shown as the mean ± SD of at least three independent experiments and the significant level was identified by ns no significance, ^**^P < 0.01, and ^***^P < 0.001.

Given these findings, we explored whether circIQGAP1 promotes tumor progression by regulating glycolytic pathways. Sphere formation assays showed ectopic circIQGAP1 increased tumor sphere size, while glycolysis inhibitor 2-DG reversed this (Fig. 3A). Similarly, 2-DG treatment abrogated the increase in cell proliferation induced by circIQGAP1 (Fig. 3B). Furthermore, migration and invasion assays showed that glycolysis inhibition eliminated the circIQGAP1-mediated increase in cell motility (Fig. 3C, D), and its promotion of colony formation was also reversed (Fig. 3E). Collectively, these results confirm circIQGAP1 promotes RCC malignant progression by activating aerobic glycolysis.

### U2AF2 induces the biogenesis of circIQGAP1

**Fig. 4:**
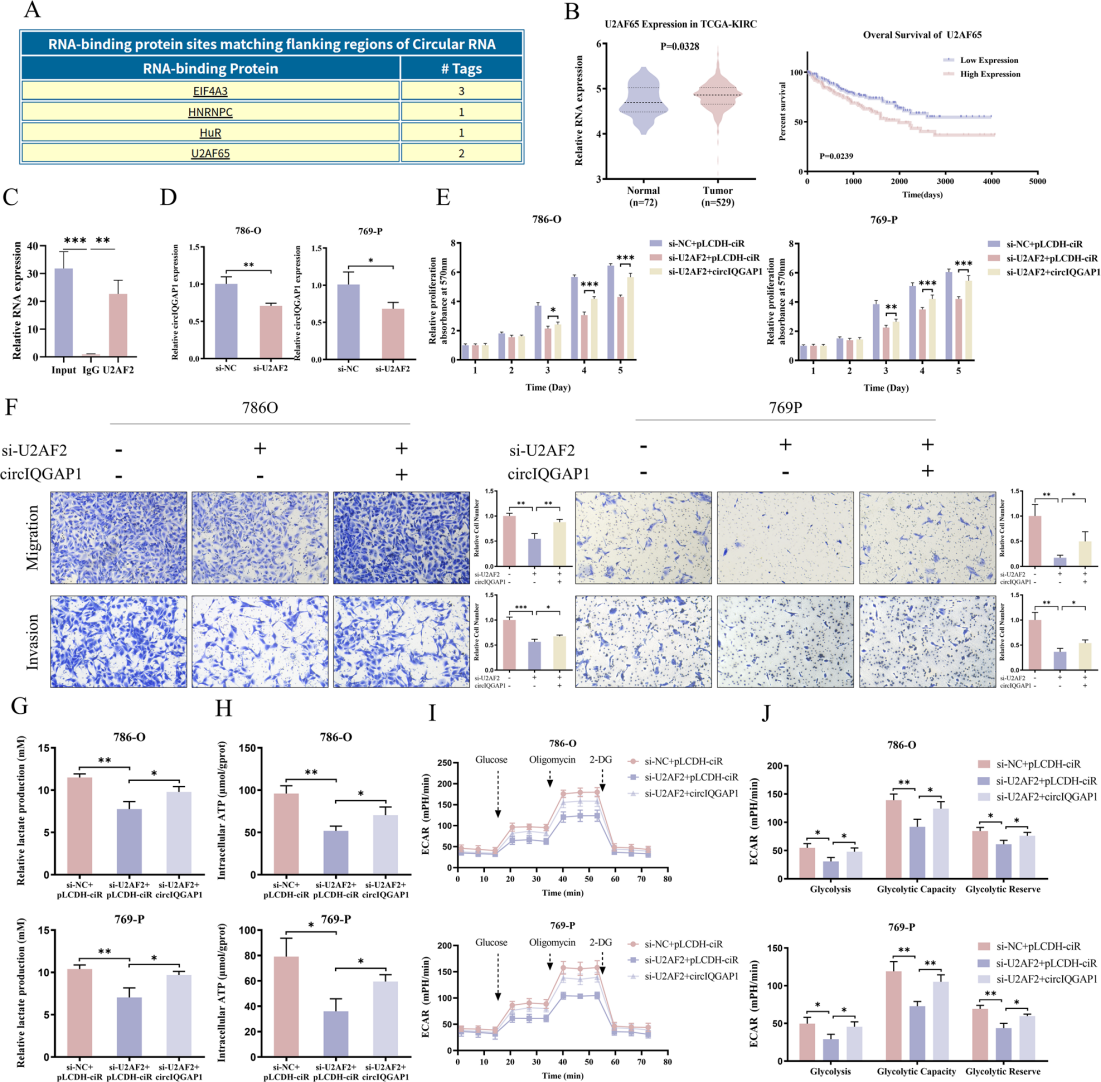
U2AF2 positively regulates circIQGAP1 to promote glycolysis and malignant. **A** Prediction of RNA-binding proteins interacting with circIQGAP1 flanking regions. **B** Analysis of U2AF2 expression in TCGA-KIRC cohort and its correlation with overall survival. **C** RNA immunoprecipitation (RIP) assay confirming the binding of U2AF2 to circIQGAP1. **D** qRT-PCR analysis of circIQGAP1 expression after U2AF2 knockout. MTT (**E**) and Transwell (**F**) assays evaluating effects of U2AF2 silencing and circIQGAP1 rescue on proliferation, migration and invasion. Lactate production (**G**) and ATP levels (**H**) after U2AF2 depletion and circIQGAP1 rescue. ECAR analysis (**I**) and quantification of basal glycolysis, glycolytic capacity, and reserve (**J**) after U2AF2 depletion and circIQGAP1 rescue. Data are shown as the mean ± SD of at least three independent experiments and the significant level was identified by ^*^P < 0.05, ^**^P < 0.01, and ^***^P < 0.001.

To explore circIQGAP1 up-regulation mechanisms, bioinformatics and experiments identified RBP U2AF65 (U2AF2) as a critical regulator (Figs. 4A and S5A-B). TCGA-KIRC data showed higher U2AF2 in tumors, correlating with shorter overall survival (OS) and enhanced proliferation (Figs. 4B and S5C). RNA immunoprecipitation (RIP) assays demonstrated specific enrichment of circIQGAP1 in samples treated with the anti-U2AF2 antibody (Fig. 4C), providing direct evidence of their physical interactions. qRT-PCR analysis further demonstrated that the knockdown of U2AF2 significantly reduced the expression level of circIQGAP1 (Fig. 4D).

To determine whether the tumor-promoting effects of U2AF2 on RCC progression are dependent on circIQGAP1, rescue experiments were performed. Exogenous circIQGAP1 expression effectively reversed the inhibitory effects of U2AF2 knockdown on cancer cell proliferation, migration, and invasion (Fig. 4E, F). Moreover, U2AF2 depletion markedly reduced lactate production and ATP generation, accompanied by decreased glycolytic activity, whereas overexpression of circIQGAP1 significantly reversed these changes (Fig. 4G–J). Taken together, these findings suggest that U2AF2 promotes aerobic glycolysis and malignant phenotypes in RCC cells by up-regulating circIQGAP1.

### CircIQGAP1 regulates CARM1 expression and stability

**Fig. 5:**
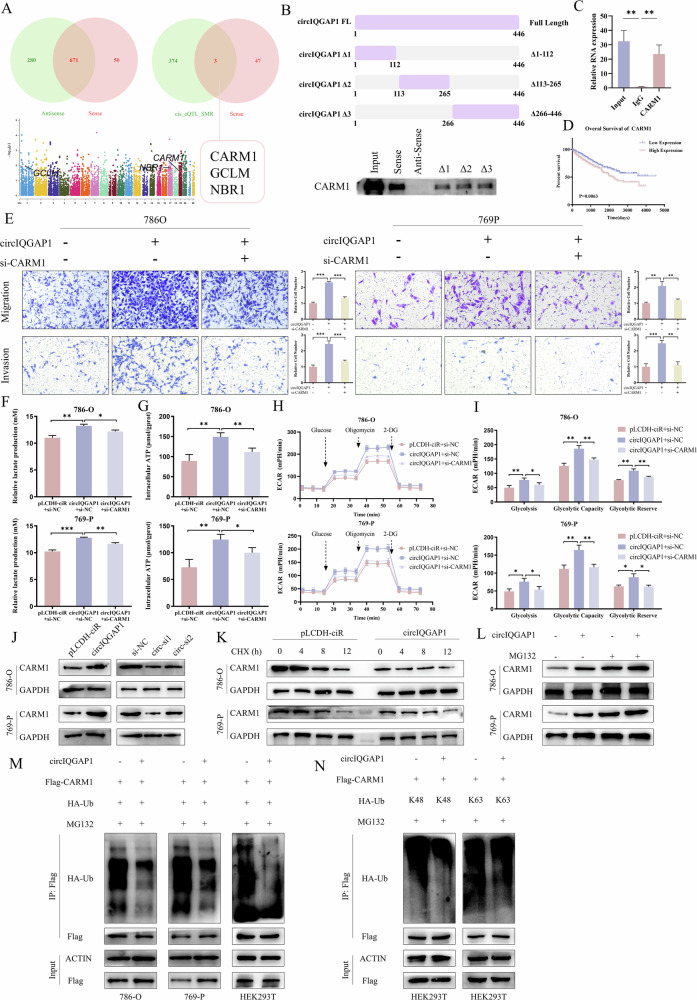
circIQGAP1 stabilizes CARM1 protein by inhibiting K48-linked ubiquitination. **A** Venn diagram (left) of circIQGAP1 pull-down candidate genes, and Venn analysis (right) with eQTL-SMR-identified RCC-associated oncogenes; Manhattan plot (right) highlights three selected proteins. **B** Interaction domain mapping between circIQGAP1 and CARM1 using wild-type and truncated biotin-labeled circIQGAP1 probes. **C** RIP confirming the interaction between CARM1 protein and circIQGAP1. **D** Kaplan–Meier survival curves stratified by CARM1 expression in RCC patients. **E** Transwell assay to evaluate migration and invasion in RCC cells co-transfected with circIQGAP1 overexpression plasmid and CARM1 siRNA. Lactate production (**F**) and ATP levels (**G**) in RCC cells co-transfected with circIQGAP1 and CARM1 siRNA. ECAR analysis (**H**) and quantification of basal glycolysis, glycolytic capacity, and reserve (**I**) after co-transfection. **J** Western blot analysis of CARM1 protein levels following circIQGAP1 modulation. **K** CHX chase assay to evaluate CARM1 half-life in RCC cells overexpressing circIQGAP1. **L** CARM1 protein levels in RCC cells overexpressing circIQGAP1 treated with proteasome inhibitor MG132. **M** Co-immunoprecipitation (Co-IP) and western blot analysis of CARM1 ubiquitination in RCC and HEK293T cells overexpressing circIQGAP1. **N** Co-IP and western blot assays to assess K48- and K63-linked polyubiquitination of CARM1 in HEK293T cell lysates. Data are shown as the mean ± SD of at least three independent experiments and the significant level was identified by ^*^P < 0.05, ^**^P < 0.01, and ^***^P < 0.001.

To clarify how circIQGAP1 regulates RCC glycolysis, RNA pull-down and mass spectrometry identified 50 circIQGAP1-binding proteins. Intersecting with 377 RCC oncogenes (eQTL-SMR) yielded three candidates: CARM1, GCLM, and NBR1 (Figs. 5A and S6A). Further experimental verification revealed that CARM1 showed a significant binding (Fig. S6B). To validate the direct interaction between circIQGAP1 and CARM1, we constructed a wild-type circIQGAP1 probe and three deletion mutants, and assessed their ability to bind to CARM1. RNA pull-down analysis showed that all three mutants had reduced binding to CARM1, suggesting that these regions are involved in the interaction, although they may not represent exclusive binding sites (Fig. 5B). Subsequent RIP analysis confirmed the specific binding between circIQGAP1 and CARM1 (Fig. 5C). Kaplan–Meier analysis indicated that high CARM1 correlated with poor RCC prognosis (Fig. 5D). Moreover, knockdown of CARM1 markedly reduced lactate production and ATP levels (Fig. S6C–E). Notably, CARM1 knockdown effectively counteracted circIQGAP1 overexpression-induced malignancy (Figs. 5E and S6F). Similarly, the increase in glycolysis by circIQGAP1 overexpression was reversed by CARM1 knockdown (Fig. 5F–I).

Further studies found that changes in circIQGAP1 expression did not affect CARM1 mRNA levels but significantly affected CARM1 protein levels (Figs. 5J and S6G). Therefore, we speculated that this change may result from post-translational regulation mediated by circIQGAP1. Cycloheximide (CHX) chase assays showed it prolonged CARM1 half-life (Fig. 5K). MG132 abolished this upregulation (Fig. 5K). In addition, treatment with the proteasome inhibitor MG132 abolished circIQGAP1-induced upregulation of CARM1 protein (Fig. 5L). Co-immunoprecipitation assays further demonstrated that circIQGAP1 overexpression significantly reduced CARM1 ubiquitination, particularly K48-linked polyubiquitination, whereas K63-linked ubiquitination was largely unaffected (Fig. 5M, N). Since K48-linked chains promote degradation [22], these findings suggest that circIQGAP1 stabilizes CARM1 by attenuating its K48-linked ubiquitination. Together, these results reveal a key role for the circIQGAP1-CARM1 axis in promoting aerobic glycolysis and driving RCC progression.

### COL5A1 functions as a key downstream effector of the circIQGAP1-CARM1 axis

**Fig. 6:**
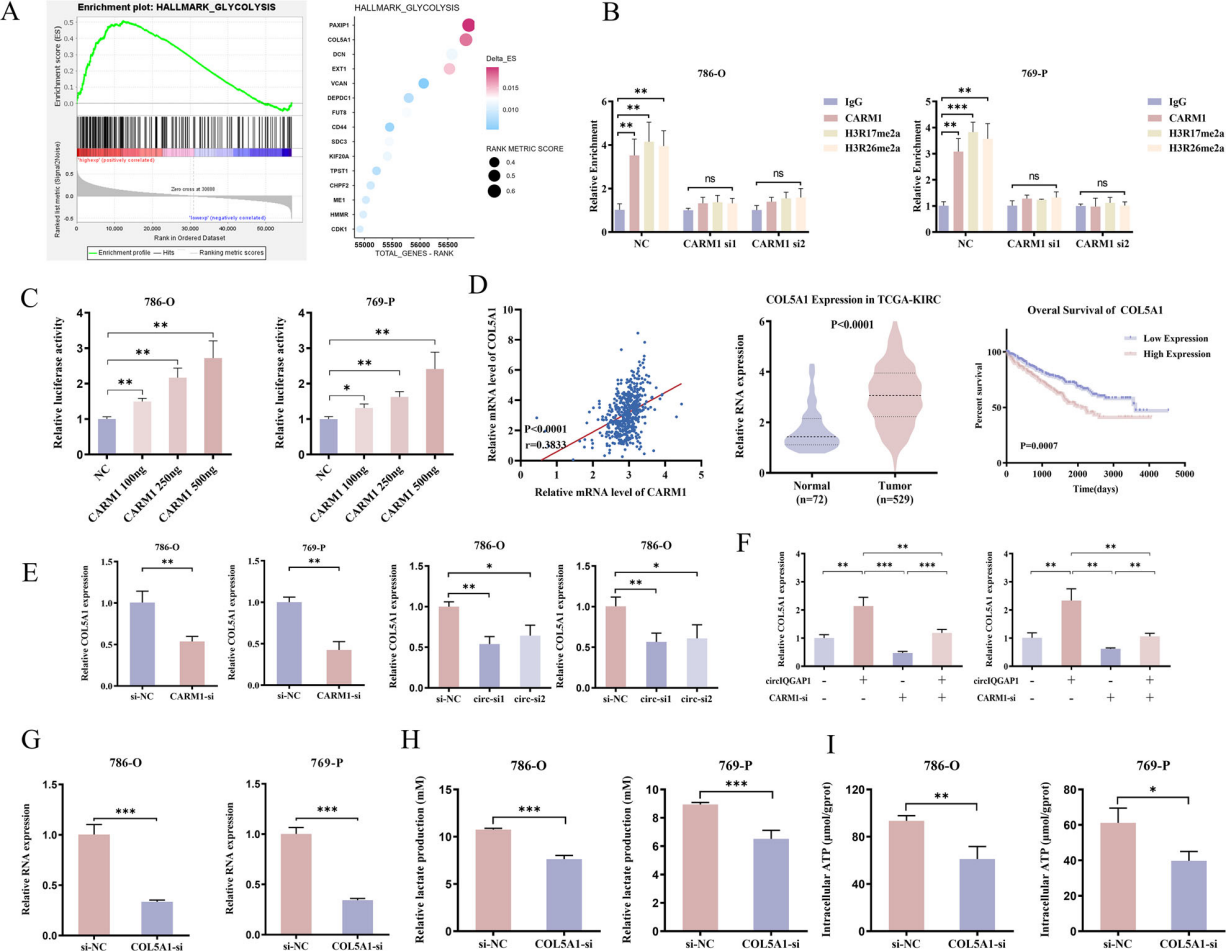
CARM1 epigenetically activates COL5A1 transcription in RCC glycolysis. **A** Gene set enrichment analysis (GSEA) plot for the HALLMARK_GLYCOLYSIS gene set upon CARM1 overexpression. **B** ChIP-qPCR analysis demonstrating enrichment of CARM1, H3R17me2a, and H3R26me2a at the COL5A1 promoter region. **C** Dual-luciferase reporter assays measuring COL5A1 promoter activity in response to increasing CARM1 expression. **D** Correlation analysis between CARM1 and COL5A1 mRNA levels, and analysis of COL5A1 expression in TCGA-KIRC cohort and its correlation with overall survival. **E** COL5A1 expression in RCC cells with CARM1 or circIQGAP1 knockdown. **F** COL5A1 expression in RCC cells co-transfected with circIQGAP1 overexpression vector and CARM1 siRNA. **G** qRT-PCR validating COL5A1 knockdown efficiency. Lactate production (**H**) and ATP levels (**I**) in RCC cells after COL5A1 knockdown. Data are shown as the mean ± SD of at least three independent experiments and the significant level was identified by ns, no significance, ^*^P < 0.05, ^**^P < 0.01, and ^***^P < 0.001.

Given that CARM1 activates transcription through histone H3 arginine 17/26 methylation [23], we further investigated its downstream transcriptional targets involved in aerobic glycolysis in RCC. GSEA revealed significant enrichment of glycolysis-related gene sets, among which PAXIP1, COL5A1, DCN, and EXT1 contributed most to the enrichment score (Fig. 6A). Subsequent qRT-PCR validation showed that only COL5A1 was consistently upregulated by circIQGAP1/CARM1 overexpression (Fig. S7A, B). To determine whether CARM1 directly regulates COL5A1 transcription, we performed chromatin immunoprecipitation (ChIP), followed by real-time PCR targeting the COL5A1 promoter region. The results demonstrated that CARM1 and H3R17me2a/H3R26me2a were significantly enriched in the COL5A1 promoter, reduced by CARM1 depletion (Fig. 6B). Dual-luciferase assays confirmed that CARM1 enhanced COL5A1 promoter activity dose-dependently (Fig. 6C). Clinically, COL5A1 expression was positively correlated with CARM1 levels, significantly elevated in tumor tissues, and its high expression predicted poor OS (Fig. 6D).

Next, we investigated whether circIQGAP1 regulates COL5A1 expression via CARM1. Silencing circIQGAP1 or CARM1 significantly suppressed COL5A1 expression (Fig. 6E). Rescue experiments showed circIQGAP1 upregulated COL5A1, reversed by CARM1 knockdown; CARM1 depletion-induced COL5A1 decrease was partially reversed by circIQGAP1 (Fig. 6F), supporting a regulatory axis in which circIQGAP1 modulates COL5A1 transcription through CARM1.

**Fig. 7:**
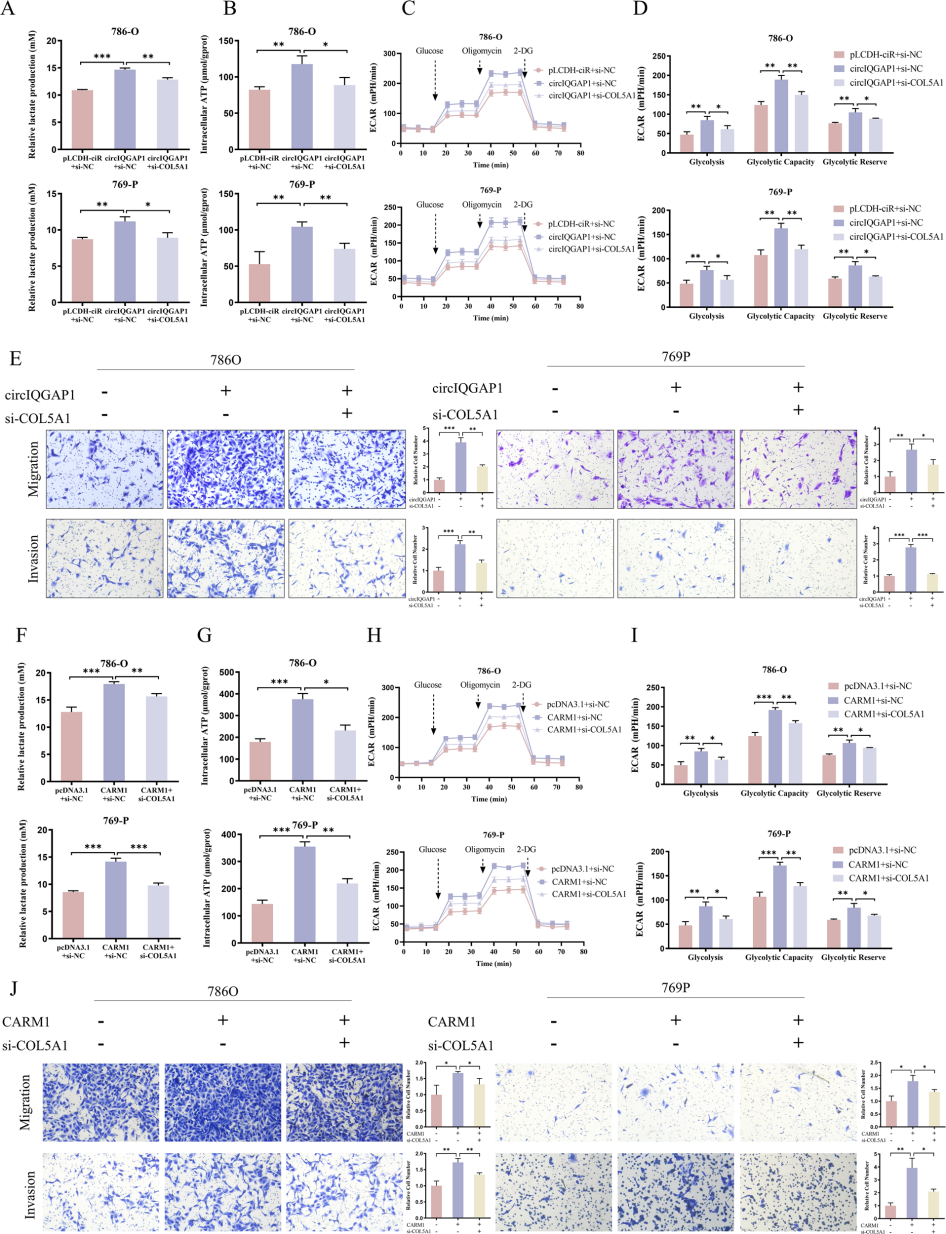
COL5A1 mediates circIQGAP1- and CARM1-driven glycolytic reprogramming and tumor progression. Lactate production (**A**) and ATP levels (**B**) in RCC cells co-transfected with circIQGAP1 overexpression plasmid and COL5A1 siRNA. ECAR analysis (**C**) and quantification of basal glycolysis, glycolytic capacity, and reserve (**D**) after co-transfection. **E** Transwell assay to evaluate migration and invasion in co-transfected cells. Lactate production (**F**) and ATP levels (**G**) in RCC cells co-transfected with CARM1 overexpression plasmid and COL5A1 siRNA. ECAR analysis (**H**) and quantification of basal glycolysis, glycolytic capacity, and reserve (**I**) in co-transfected cells. **J** Transwell assay to evaluate migration and invasion in RCC cells co-transfected with CARM1 overexpression plasmid and COL5A1 siRNA. Data are shown as the mean ± SD of at least three independent experiments and the significant level was identified by ^*^P < 0.05, ^**^P < 0.01, and ^***^P < 0.001.

Functionally, COL5A1 silencing not only reduced lactate production and ATP generation (Fig. 6G–I) but also completely abrogated the glycolytic enhancement induced by circIQGAP1 (Fig. 7A–D). Notably, COL5A1 deletion reversed circIQGAP1-mediated oncogenic phenotypes, including enhanced proliferation, migration, and invasion (Figs. 7E and S7C). Similar effects were seen in CARM1-overexpressing cells (Figs. 7F–J and S7D). Thus, COL5A1 is the key downstream effector of the circIQGAP1-CARM1 axis in RCC.

### circIQGAP1 drives RCC progression in vivo and holds biomarker potential

**Fig. 8:**
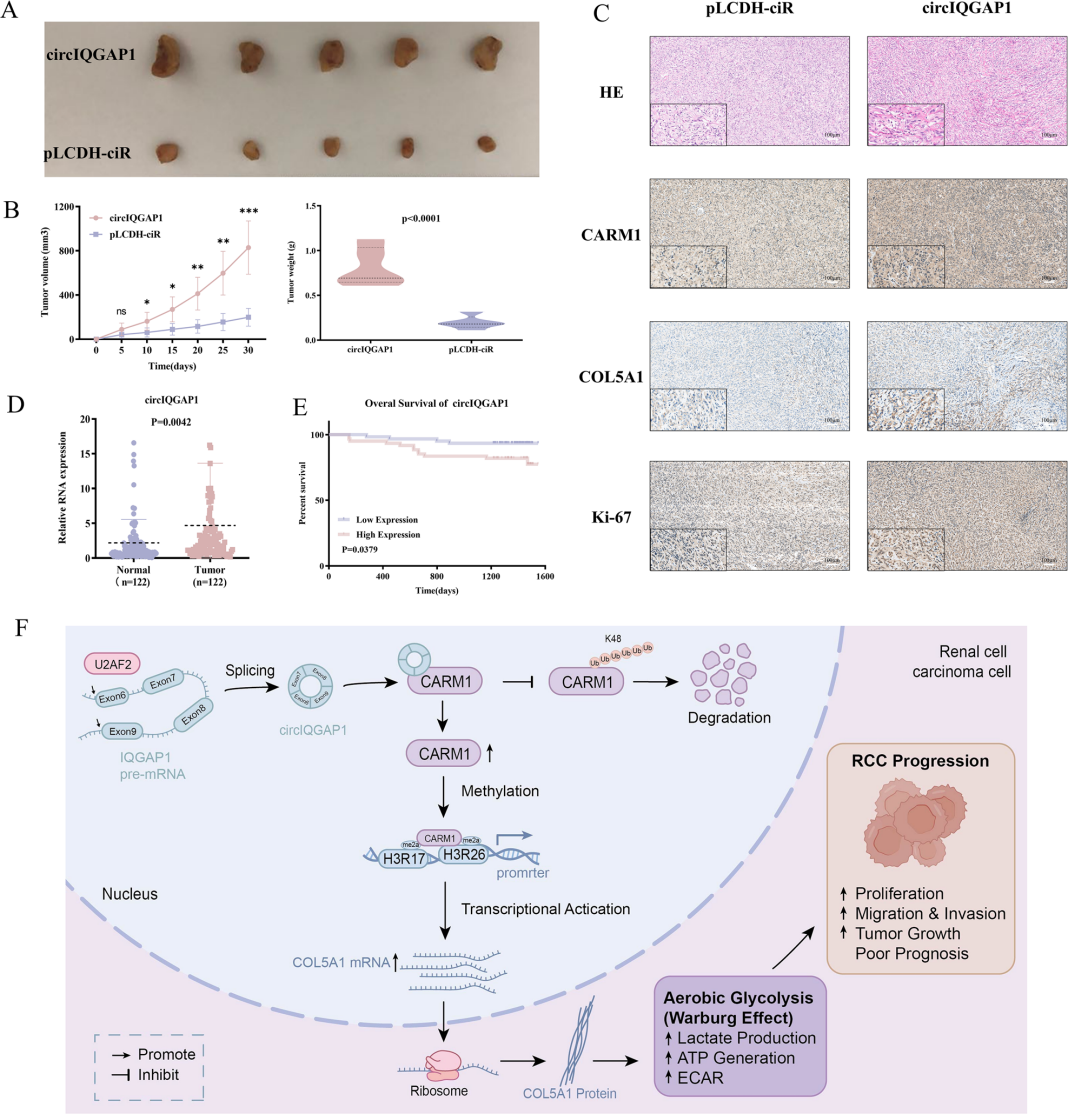
In vivo tumorigenesis and clinical correlation of circIQGAP1 in RCC. **A** Representative images of xenograft tumors formed by circIQGAP1-overexpressing or control RCC cells in mice. **B** Tumor volume growth curves and final tumor weights after 30 days in two groups of mice. **C** Representative images of HE staining (tumor histology) and IHC (CARM1, COL5A1, and Ki67) in xenograft tumor tissues from two groups. **D** circIQGAP1 expression levels in plasma of RCC patients and healthy individuals. **E** Kaplan–Meier survival curves of RCC patients stratified by circIQGAP1 expression. **F** Schematic illustration of the circIQGAP1-CARM1 axis promoting RCC progression via enhanced glycolysis. Data are shown as the mean ± SD of at least three independent experiments, and the significant level was identified by ns, no significance, ^**^P < 0.01, and ^***^P < 0.001.

To investigate the in vivo role of circIQGAP1 in RCC progression, we established a xenograft mouse model using RCC cells transfected with circIQGAP1 vectors or a control plasmid pLCDH-ciR. Compared to control mice, mice injected with cells overexpressing circIQGAP1 exhibited markedly accelerated xenograft tumor growth, with significant increases in tumor volume and weight (Fig. 8A, B). HE and IHC analyzes further showed that the expression of CARM1, COL5A1, and the proliferation marker Ki67 was elevated in circIQGAP1-derived tumors (Fig. 8C). Plasma sample analysis of patients with RCC and healthy individuals demonstrated that circIQGAP1 levels were significantly increased in the patient group (Fig. 8D). Kaplan–Meier survival analysis further demonstrated that higher circIQGAP1 expression was significantly associated with poorer prognosis in patients with RCC (Fig. 8E). Collectively, these results suggest that circIQGAP1 promotes tumor growth in vivo (Fig. 8F) and may serve as a diagnostic and prognostic biomarker for RCC.

## Discussion

As a typical tumor metabolic feature, the Warburg effect allows cancer cells to convert glucose to lactate under normoxia, fueling rapid ATP production and macromolecule synthesis needed for their proliferation and survival [24]. Lactate accumulation not only acidifies the tumor microenvironment, promoting invasion and metastasis, but also inhibits antitumor immune responses by inhibiting cytotoxic T and natural killer cells [25]. Moreover, tumor-associated stromal cells and immune infiltration undergo glycolytic reprogramming, further driving tumor progression and treatment resistance [26]. RCC is a metabolically highly active solid tumor, and its malignant progression is closely related to the abnormal activation of aerobic glycolysis [27]. Advanced RCC with high-grade or metastatic features is often accompanied by metabolic reprogramming toward glycolysis, which correlates with poor clinical outcomes [28]. Recent studies have highlighted the key role of circRNAs in the regulation of glucose metabolism in RCC. For example, circVAMP3 can enhance aerobic glycolysis and proliferation of RCC cells by binding to and promoting the activation of the glycolytic enzyme LDHA [29]; circME1 can upregulate ME1 gene expression by interacting with U1 snRNP, thereby promoting glucose metabolism and sunitinib resistance in ccRCC cells [30]. In this study, we identified circIQGAP1 as a new circRNA that promotes RCC glycolysis, which is significantly upregulated under low-glucose conditions, and verified its key role in the malignant phenotype.

We further found that circIQGAP1 was upregulated by U2AF2. As a component of the spliceosome RBP, U2AF2 can accurately recognize the 3′-terminal uracil-rich sequence of pre-mRNA and recruit U2 snRNP to assemble the splicing complex [31]. Previous studies have shown that RBPs play important roles in the RNA splicing process, not only by participating in linear RNA splicing, but also by generating oncogenic circRNAs through back-splicing [32, 33]. Studies on gliomas have shown that U2AF2 can promote tumor cell proliferation, angiogenesis, and malignant phenotypes by promoting the biogenesis of circARF1 and circNCAPG [34, 35]. Our findings extend this paradigm to RCC, indicating that U2AF2 binds to the flanking sequence motif of circIQGAP1 to promote its circularization.

Further studies revealed that circIQGAP1 specifically binds to CARM1 through a multi-domain cooperative mode, thereby enhancing the stability of the protein. Previous studies have shown that circRNAs can protect target proteins from degradation by the ubiquitin-proteasome system by blocking protein ubiquitination. For example, circMTCL1 and circ_0084653 inhibit ubiquitination degradation of C1QBP and MYC proteins, respectively, thereby promoting cancer progression [36, 37]. In addition, a study on liver cancer found that the CARM1 protein is regulated by ubiquitination, and PSMD14 stabilizes CARM1 by reducing its ubiquitination, consequently promoting the proliferation and metastasis of liver cancer [38]. Collectively, these findings suggest that circIQGAP1 may use a similar mechanism to stabilize CARM1. Consistent with this hypothesis, our data showed that circIQGAP1 reduced the K48-linked polyubiquitination of CARM1, significantly prolonged its half-life and maintained elevated protein levels.

CARM1 is overexpressed in various malignancies, including gastric cancer [39], colorectal cancer [40], breast cancer [41, 42], ovarian cancer [43], and osteosarcoma [44]. In these tumors, it functions as a powerful oncogenic driver. CARM1, a protein arginine methyltransferase, catalyzes histone and non-histone methylation events, reprogramming tumor metabolism and transcriptional networks [45, 46]. Specifically, CARM1-mediated methylation of PKM2 shifts cellular metabolism to aerobic glycolysis, while inhibiting mitochondrial oxidative phosphorylation, thereby driving the Warburg phenotype of tumor cells [47, 48]. In addition, CARM1 methylates O-GlcNAc transferase and GAPDH, further regulating glycolytic flux and adapting cancer cells to fluctuating glucose levels [49, 50]. In addition to metabolic enzymes, CARM1 also supports cell proliferation and metastasis by methylating key proteins like NF-κB and p53 or regulating chromatin structure [51–53]. Therefore, upregulation of CARM1 by circIQGAP1 may provide a way for RCC to promote a malignant phenotype by regulating energy metabolism.

Through multi-omics analysis and functional validation, we identified COL5A1 as a key transcriptional target gene of CARM1 in the glycolytic pathway. CARM1 significantly activates COL5A1 transcription by directly binding to its promoter and inducing histone H3 methylation. As an important low-abundance fibrillar collagens, COL5A1 participates in extracellular matrix (ECM) remodeling and is closely associated with tumor proliferation, migration, and invasion [54]. In gastric cancer models, COL5A1 has been confirmed to enhance cell migration and epithelial-mesenchymal transition (EMT) [55]. In ccRCC, its high expression is significantly associated with tumor metastasis and reduced patient survival [56, 57]. Multiple bioinformatics studies have identified COL5A1 as a glycolysis-related gene [58, 59]. In glioblastoma, NOX2 upregulates COL5A1 expression by enhancing glucose metabolic activity, further promoting mesenchymal transformation of tumor cells, suggesting that COL5A1 may promote tumor metabolic reprogramming and EMT [60]. A stiff ECM can induce intracellular glycolysis [61], and COL5A1, as a key component of the ECM, may play a role in this process. Notably, its circRNA variant circCOL5A1 has been shown to drive aerobic glycolysis and tumor progression in RCC [62]. In our study, COL5A1 gene knockout not only reversed the enhanced glucose metabolism mediated by circIQGAP1 and CARM1 but also significantly inhibited the corresponding malignant phenotype. However, the specific downstream regulatory mechanism of COL5A1 needs to be further studied to provide more precise molecular targets for targeting this pathway.

In summary, this study systematically revealed the core role of circIQGAP1 in driving aerobic glycolysis and malignant progression of RCC through CARM1-dependent epigenetic reprogramming. Notably, the expression level of circIQGAP1 in the plasma of patients with RCC was significantly higher than that in healthy individuals, indicating its potential as a diagnostic biomarker. Functional experiments showed that loss of CARM1 function effectively reversed the glycolytic enhancement effect driven by circIQGAP1. As a key node in the cellular metabolic regulatory network, targeted intervention against CARM1 has demonstrated clear potential in cancer therapy [63]. Early inhibitors EZM2302 and TP-064 have been shown to inhibit tumor proliferation in multiple cancer models, including multiple myeloma and endometrial cancer [64, 65].

Beyond metabolic regulation, CARM1 is involved in immune regulation in the tumor microenvironment. Suppression of CARM1 enhances infiltration by CD8+ T cells, natural killer cells, and dendritic cells while simultaneously reducing the proportion of CD8+ T cells expressing inhibitory PD-1 and TIM-3 receptors [66]. CARM1-deficient T cells display augmented tumor penetration and a memory phenotype associated with durable antitumor immunity [66]. Additionally, CARM1 inhibition can induce tumor cells to produce a type 1 interferon response, significantly improving the efficacy of immunotherapy, and this effect is particularly evident in immunotherapy-resistant populations [66, 67]. These findings suggest that CARM1 holds promise as a strategic combinatorial target for overcoming immunotherapy resistance in RCC. In preclinical models of PD-L1 resistance, CARM1 suppression epigenetically activates the cGAS-STING axis, promotes dendritic cell maturation, enhances T cell priming, and upregulates CXCR3 expression to facilitate T cell recruitment [68]. In non-small cell lung cancer, research has found that relieving CARM1 suppression deactivates type I interferon responses to promote immune escape, and crucially, the combination of CARM1 inhibitors and PD-1 blockade has been shown to enhance tumor sensitivity to immunotherapy [67]. Supporting this, clinical data reveal that RCC patients who respond to PD-L1 blockade have significantly lower tumor CARM1 expression than non-responders [66]. Furthermore, investigations on osteosarcoma have shown that the inhibition of CARM1 can control aerobic glycolysis, which is also crucial for overcoming tumor drug resistance [69]. Collectively, these data suggest that the strategy of CARM1 inhibition combined with immunotherapy may have the potential to provide an innovative solution for reversing drug resistance in RCC.

## Supplementary information

Supplementary Figure

uncropped Gels and Blots images

## Data Availability

The data underlying the results presented in this paper are available from the corresponding author upon reasonable request. Full and uncropped blots are provided with this paper (Supplemental Material).
