# Supplementary material for: CircIQGAP1-CARM1 axis promotes renal cell carcinoma progression through glycolytic reprogramming
Source: Cell Death Dis. 2026 Mar 27;17(1):414. doi: 10.1038/s41419-026-08661-w (PMC13144507; doi:10.1038/s41419-026-08661-w)
Supplement: Supplementary file 1 — Supplementary Figure [file 41419_2026_8661_MOESM1_ESM.pdf]

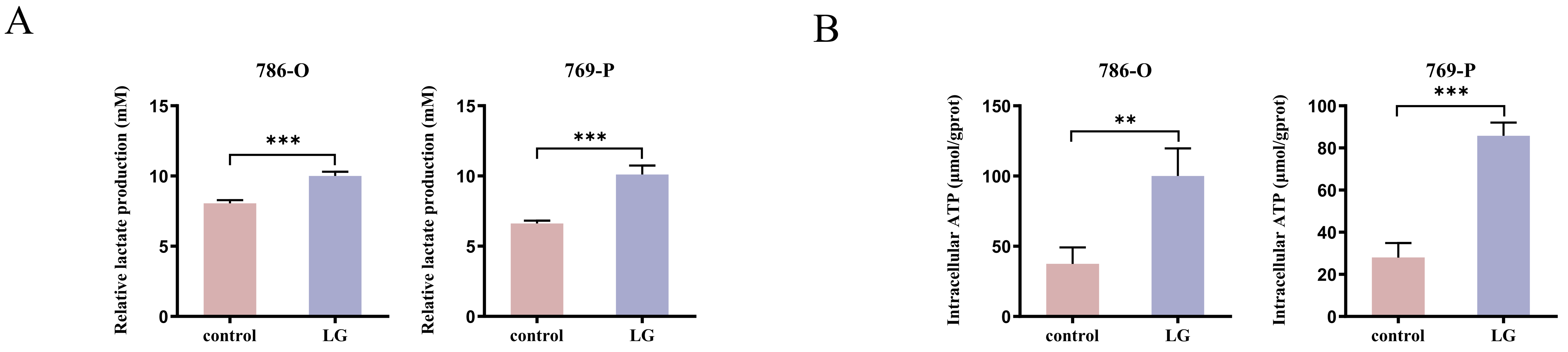


**Supplementary Figure 1 Enhanced glycolysis in renal cancer cells under low glucose treatment.** (A, B) Lactate secretion (A) and intracellular ATP levels (B) in renal carcinoma cells after low glucose treatment.

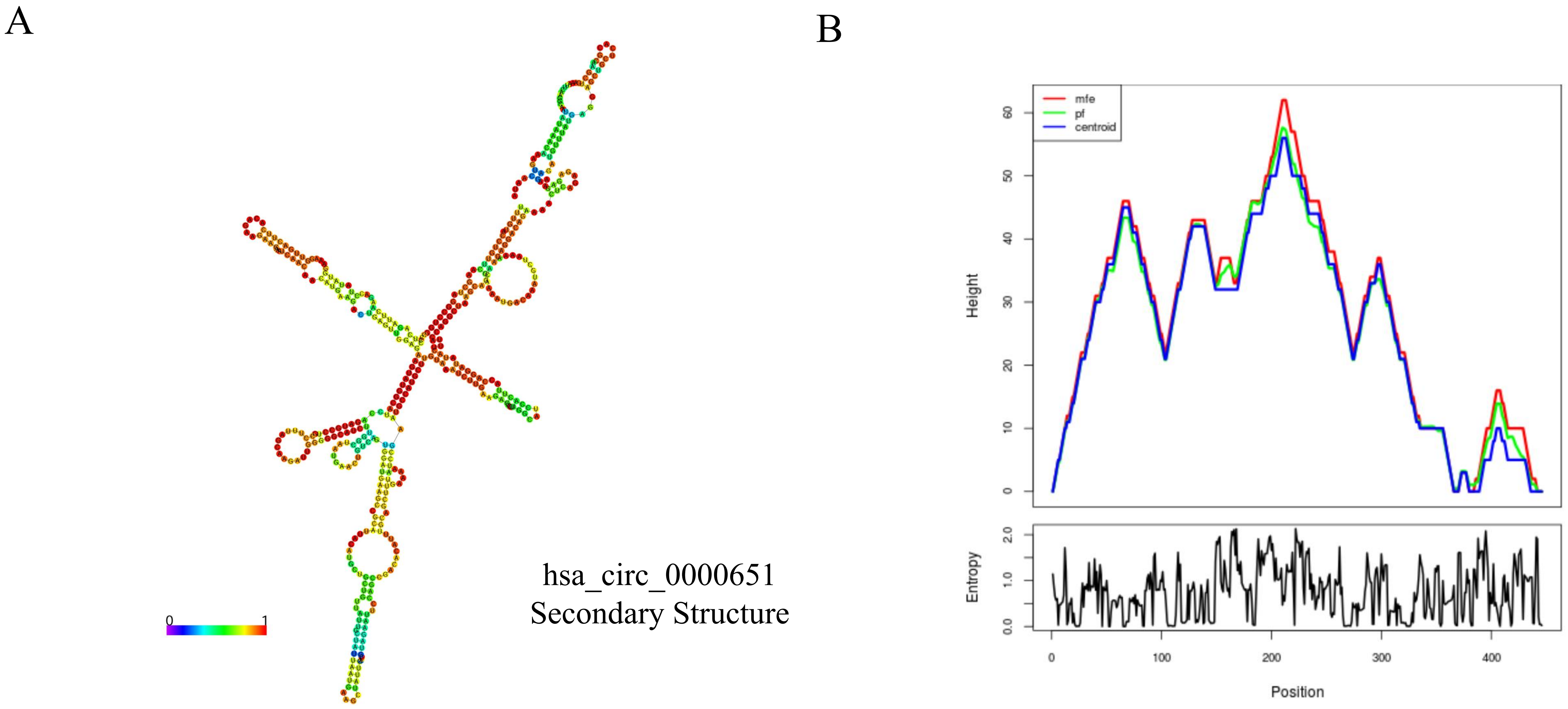


**Supplementary Figure 2 Secondary structure prediction and structural parameter analysis of circIQGAP1.** (A) Predicted secondary structure of circIQGAP1 showing its complex folding pattern. (B) Base pairing stability (top) and conformational diversity at each position (bottom) of circIQGAP1. Curves in different colors represent predictions from distinct algorithms or parameter settings.

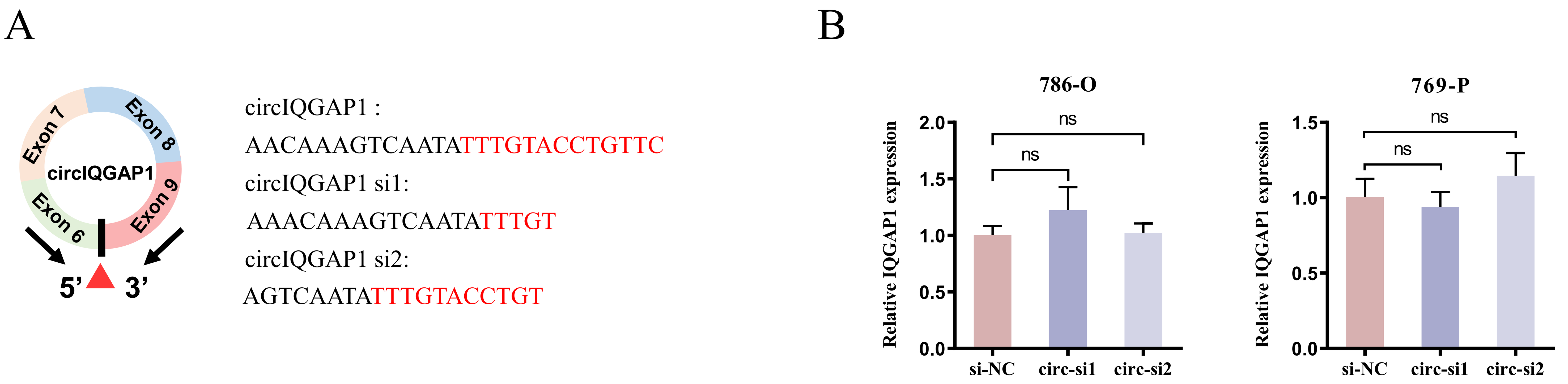


**Supplementary Figure 3 Sequences of circIQGAP1-specific siRNAs and specificity validation.** (A) Sequences of two siRNAs targeting the back-splicing junction of circIQGAP1. (B) qRT-PCR analysis confirming circIQGAP1 knockdown didn't affect linear IQGAP1 mRNA levels.

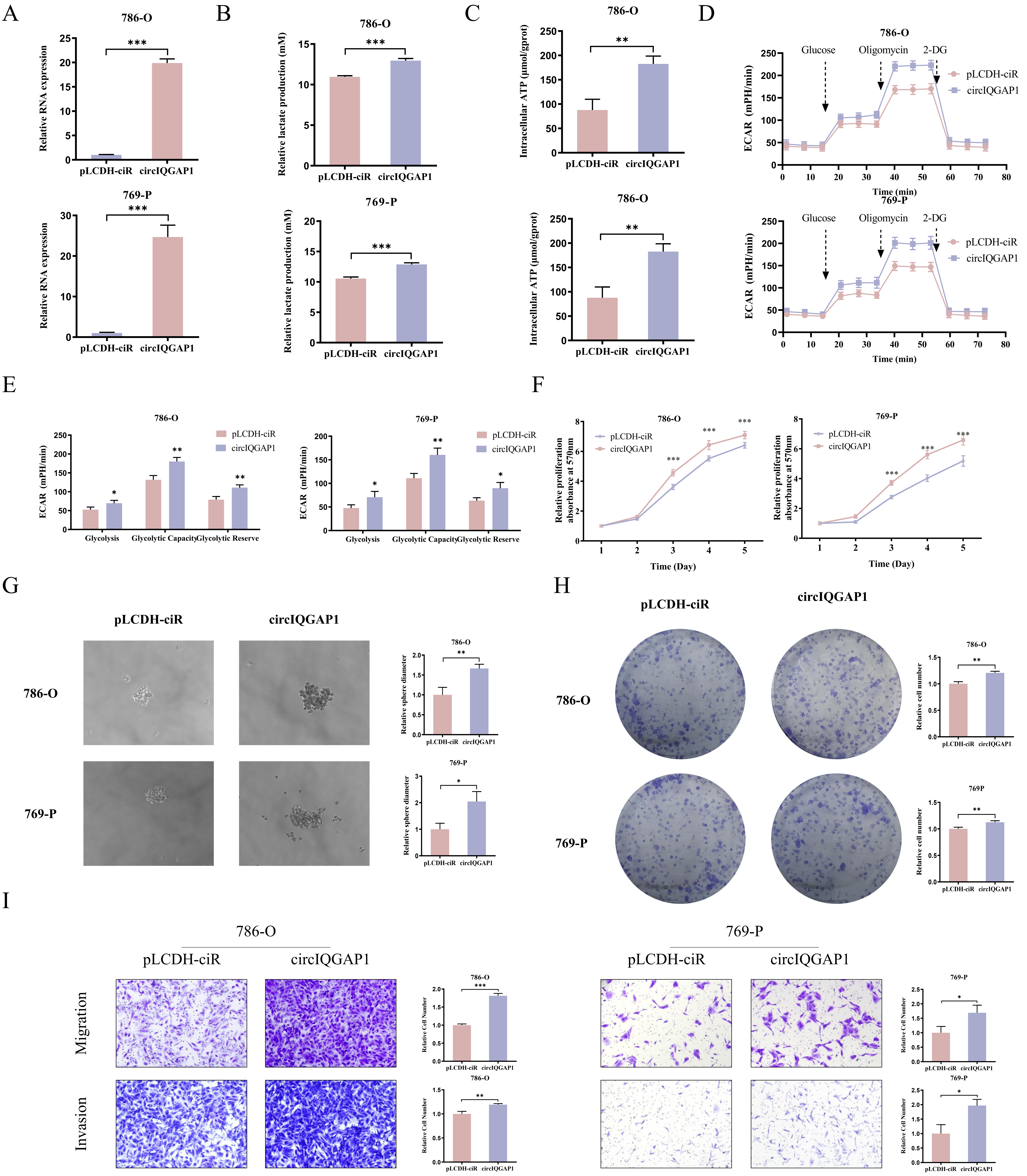

A
786-O
769-P
Relative RNA expression
***
pLCDH-ciR
circIQGAP1
B
Relative lactate production (mM)
C
Intracellular ATP (μmol/gprot)
**
D
ECAR (mPH/min)
Glucose
Oligomycin
2-DG
Time (min)
E
Glycolysis
Glycolytic Capacity
Glycolytic Reserve
F
Relative proliferation absorbance at 570nm
Time (Day)
G
Relative sphere diameter
H
Relative cell number
769P
I
Migration
Invasion
Relative Cell Number

**Supplementary Figure 4 Functional validation of circIQGAP1 overexpression in glycolysis regulation and malignant behaviors.** (A) qRT-PCR validation of circIQGAP1 overexpression efficiency in renal cancer cells transfected with pLCDH-ciR or circIQGAP1-overexpressing plasmids. (B, C) Lactate production (B) and ATP levels (C) in renal cancer cells overexpressing circIQGAP1. (D, E) Extracellular acidification rate (ECAR) (D) of renal cancer cells overexpressing circIQGAP1, and the glycolytic parameters (basal glycolysis, glycolytic capacity, and glycolytic reserve) (E) were evaluated. (F-I) MTT assay (F), tumor spheroid formation (G), colony formation (H), and Transwell assay (I) used to evaluate proliferation, migration, and invasion in renal cancer cells overexpressing circIQGAP1.

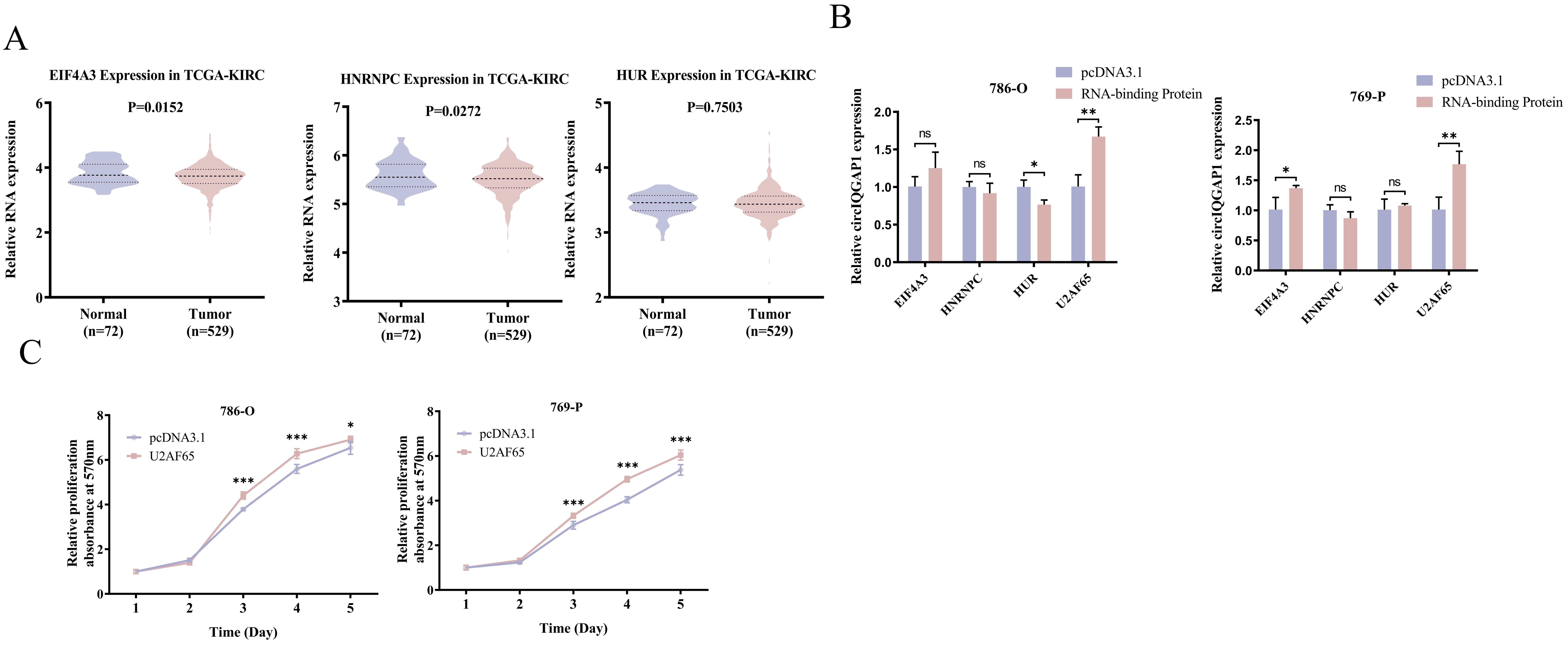


**Supplementary Figure 5 Identification of RNA-binding proteins interacting with circIQGAP1.** (A) Expression levels of RNA-binding proteins interacting with circIQGAP1 in the TCGA-KIRC cohort. (B) qRT-PCR analysis of the effect of candidate RNA-binding proteins on circIQGAP1 expression. (C) MTT assay assessing proliferation capacity of renal cancer cells overexpressing U2AF65 (U2AF2).

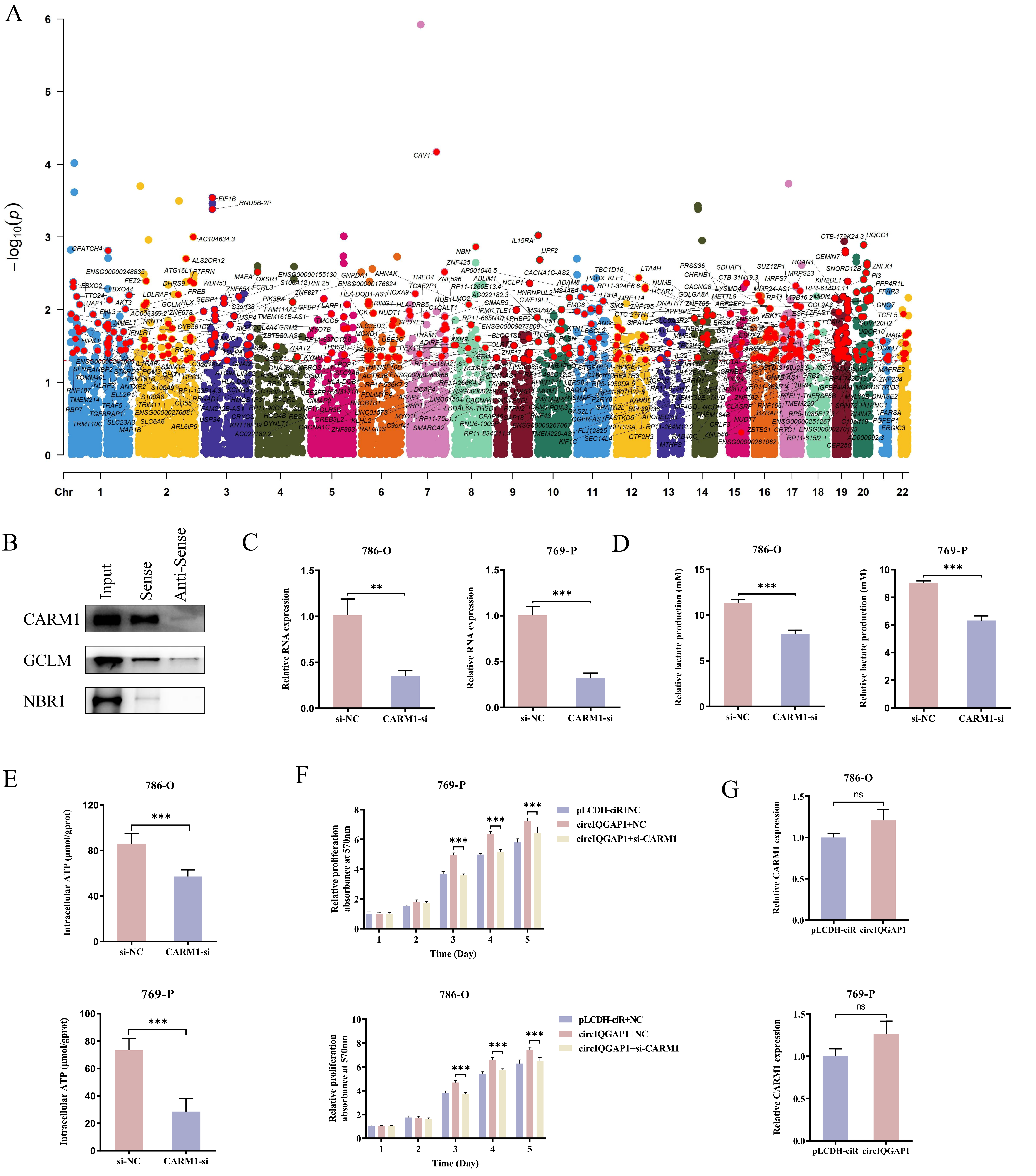


**Supplementary Figure 6 Validation of candidate proteins interacting with circIQGAP1.** (A) eQTL-SMR analysis identifying differentially expressed genes between RCC and normal samples, with oncogenes highlighted in red. (B) RNA pull-down assay to detect the binding of circIQGAP1-binding proteins to circIQGAP1. (C) qRT-PCR validation of CARM1 expression in RCC cells transfected with CARM1 siRNA. (D, E) Lactate production (D) and ATP levels (E) in RCC cells transfected with CARM1 siRNA. (F) MTT assay to evaluate proliferation in RCC cells co-transfected with circIQGAP1 overexpression plasmid and CARM1 siRNA. (G) The mRNA level of CARM1 was detected by qRT-PCR in RCC cells overexpressing circIQGAP1.

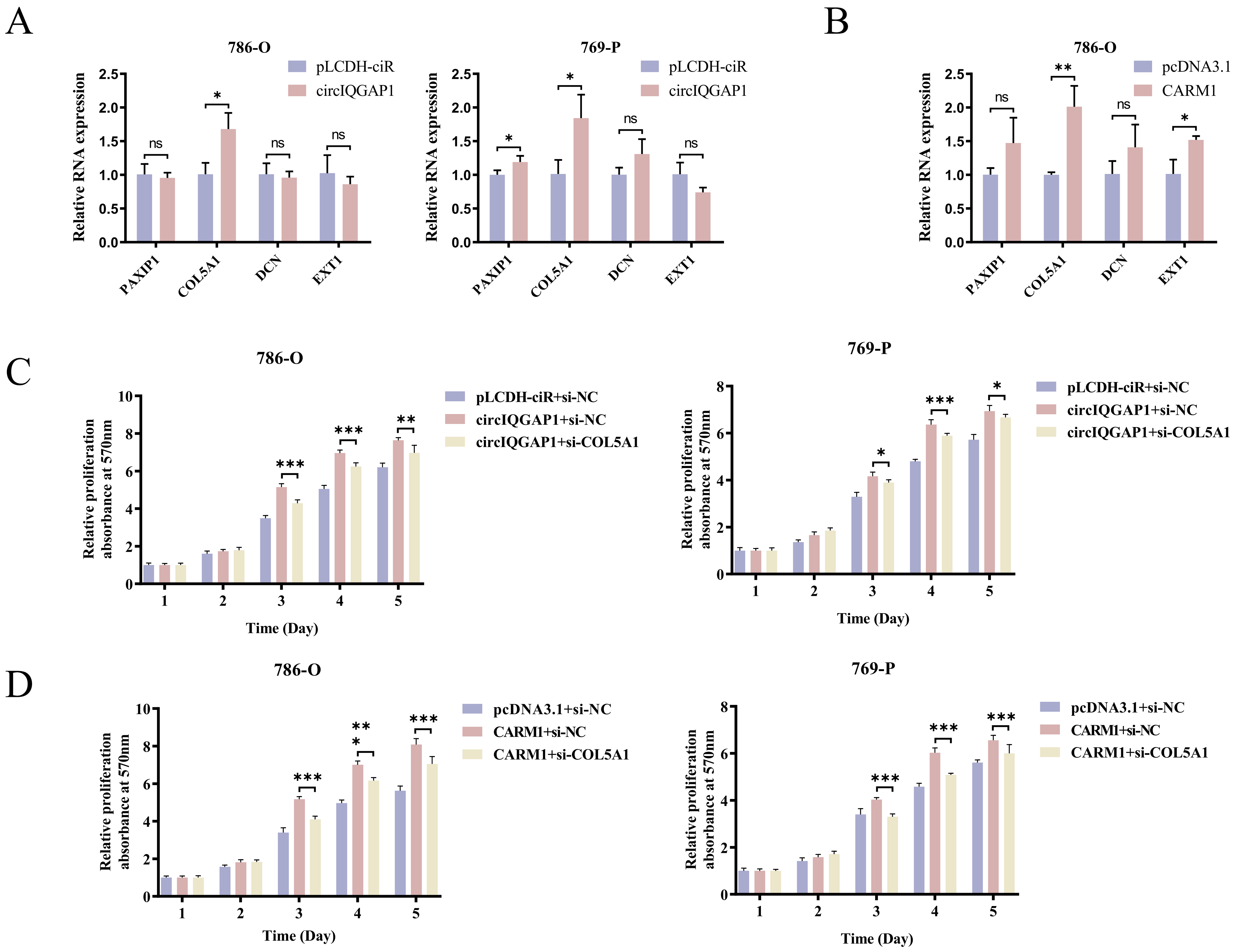


**Supplementary Figure 7 Validation of COL5A1 as a glycolysis-related transcriptional target of the circIQGAP1-CARM1 axis.** (A, B) qRT-PCR analysis of glycolysis-related genes in RCC cells overexpressing circIQGAP1 (A) or CARM1 (B). (C, D) MTT assay evaluating proliferation capacity of RCC cells co-transfected with circIQGAP1 (C) or CARM1 (D) overexpression plasmids and COL5A1 siRNA.
