# Supplementary figures and images for: CircIQGAP1-CARM1 axis promotes renal cell carcinoma progression through glycolytic reprogramming
Source: Cell Death Dis. 2026 Mar 27;17(1):414. doi: 10.1038/s41419-026-08661-w (PMC13144507; doi:10.1038/s41419-026-08661-w)

**Figure 1F**

**786-O**

**769-P**

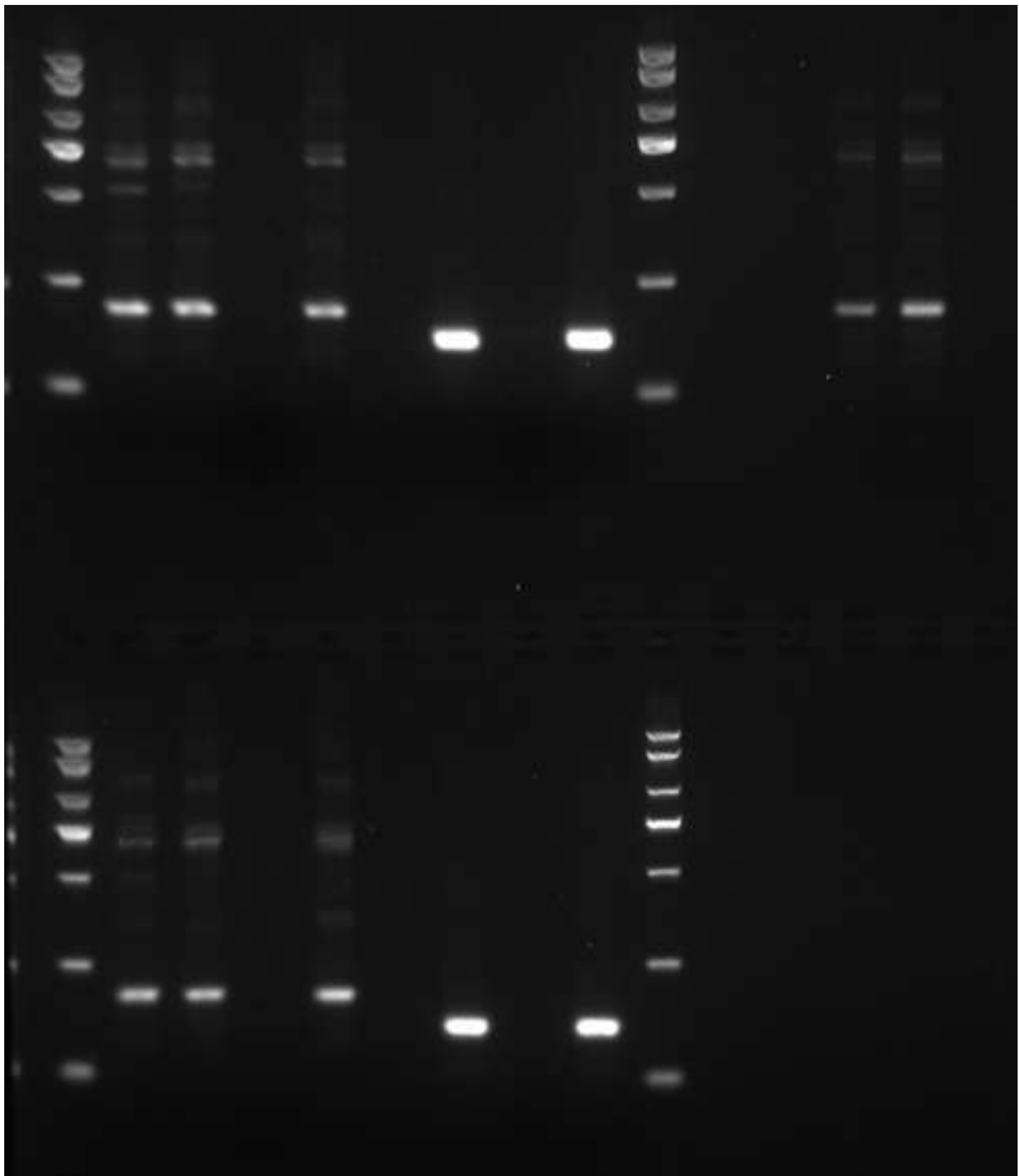

**Figure 5B**

**CARM1**

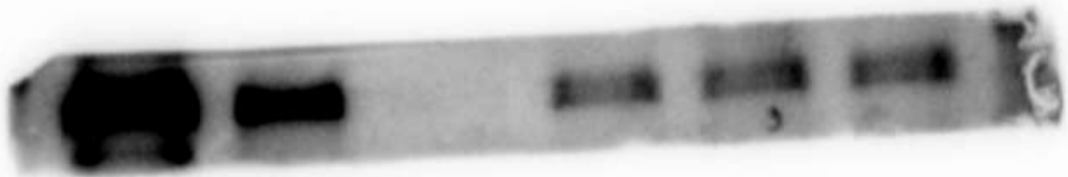

Figure 5J

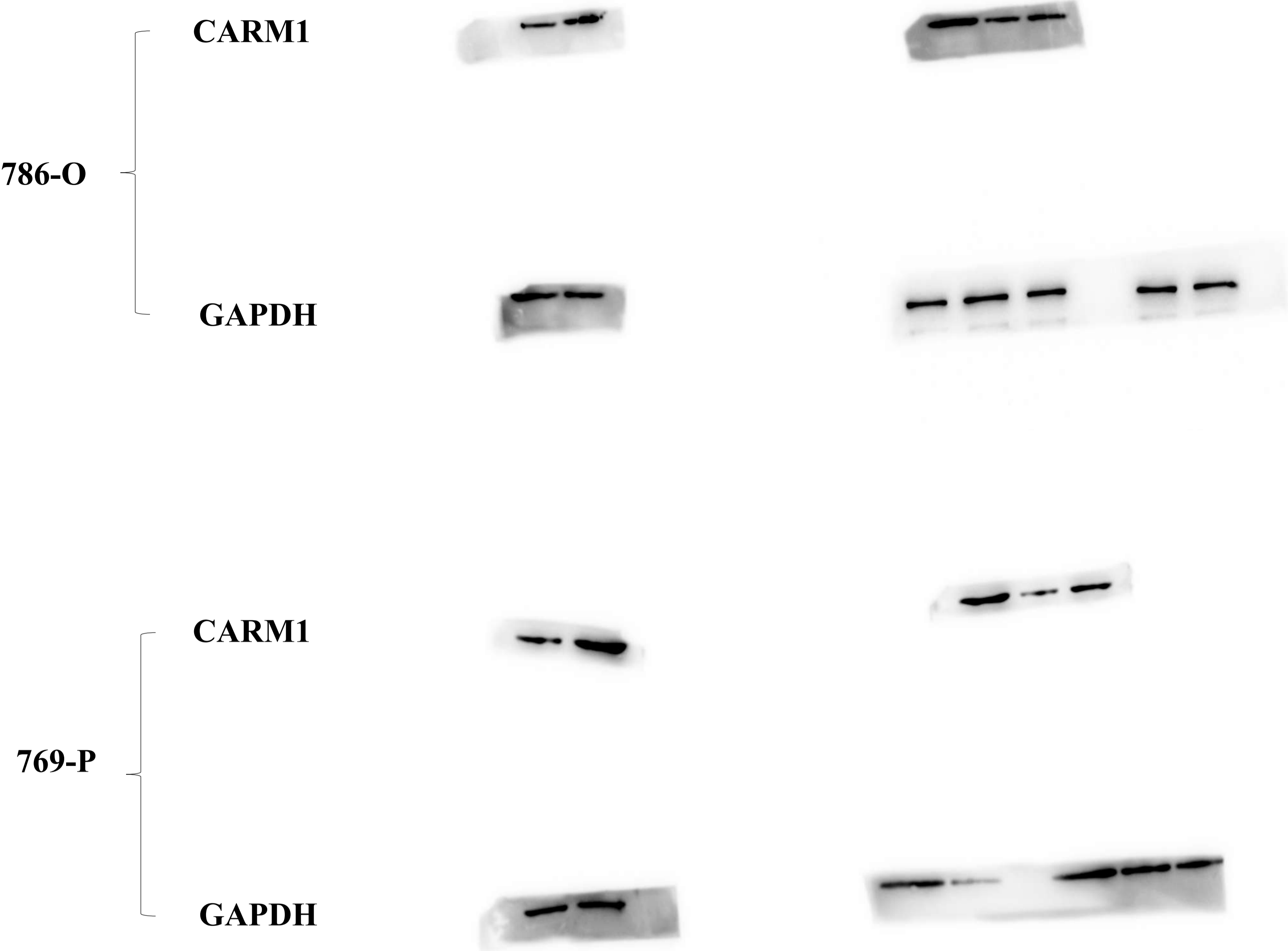

Figure 5K

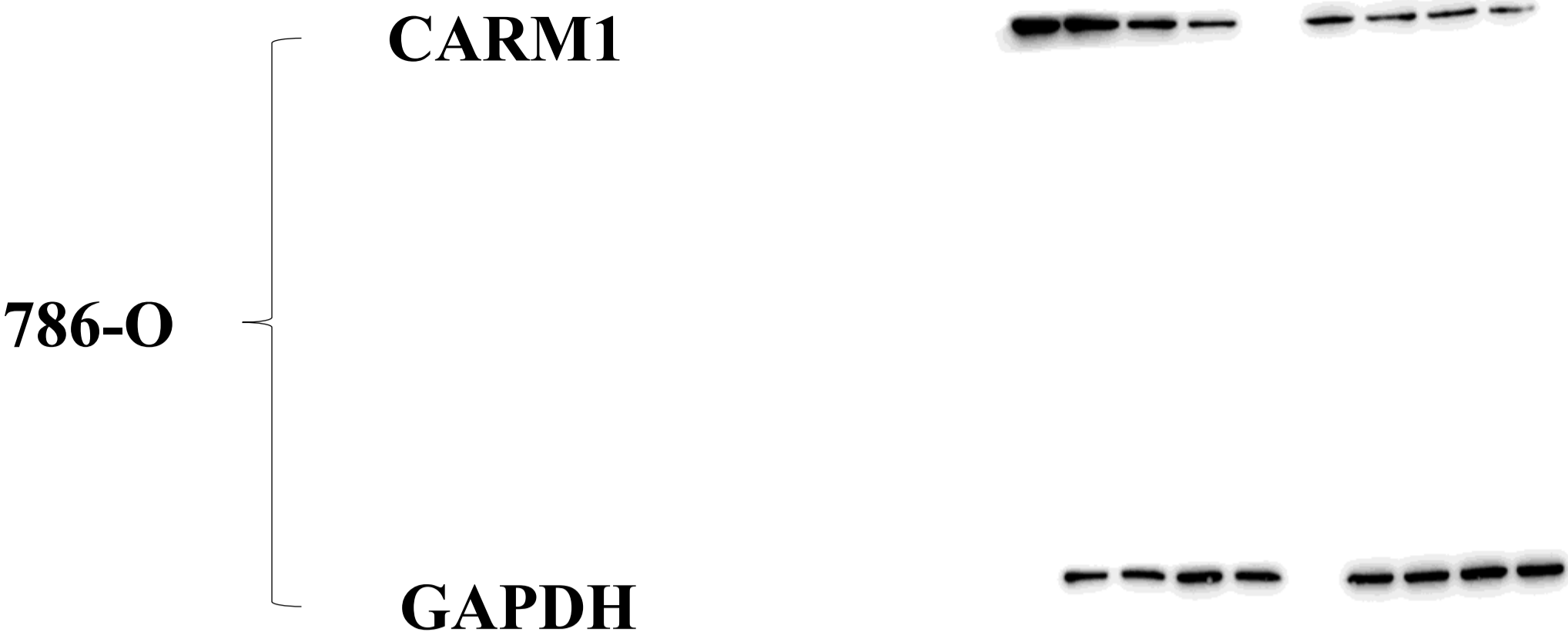

769-P

CARM1

GAPDH

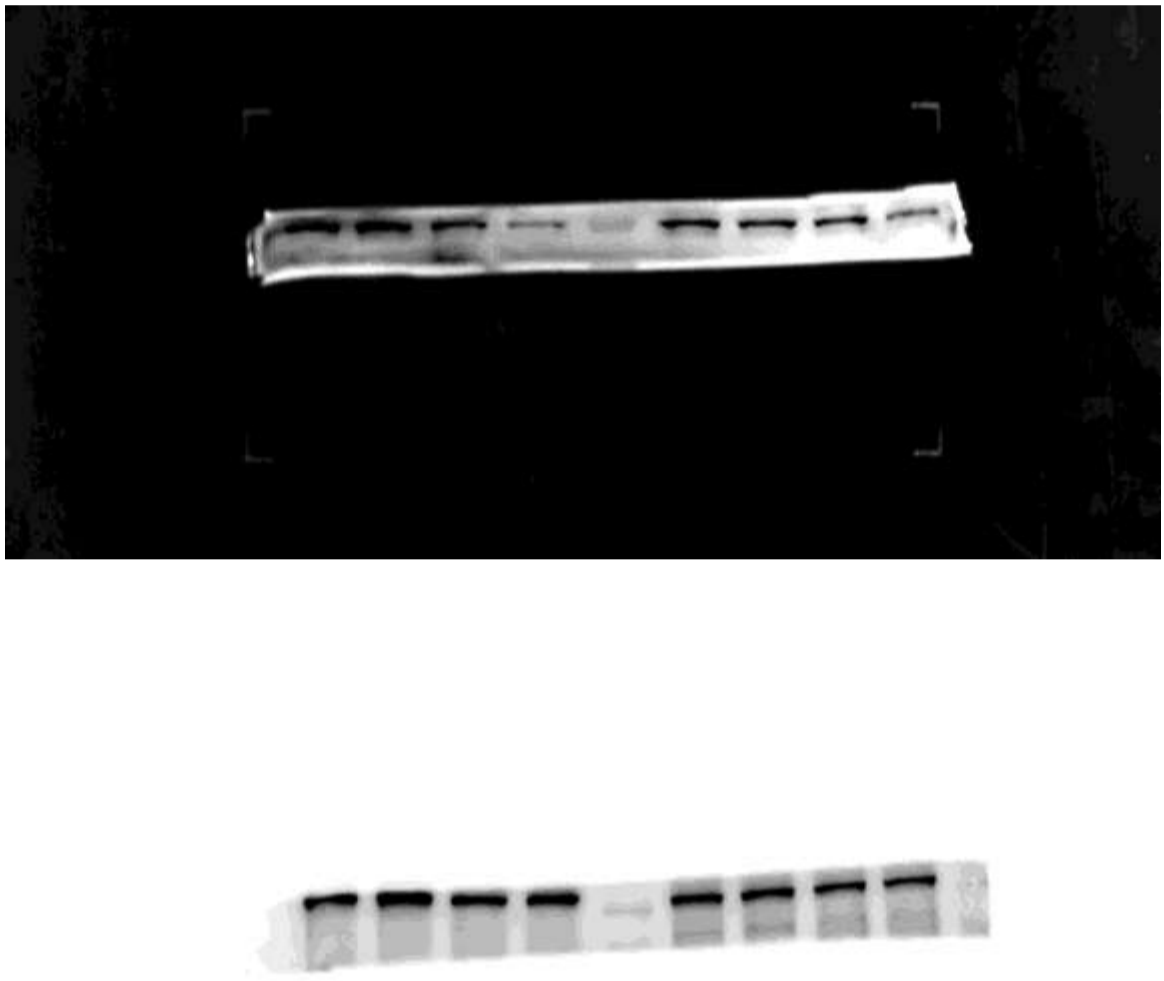

Figure 5L

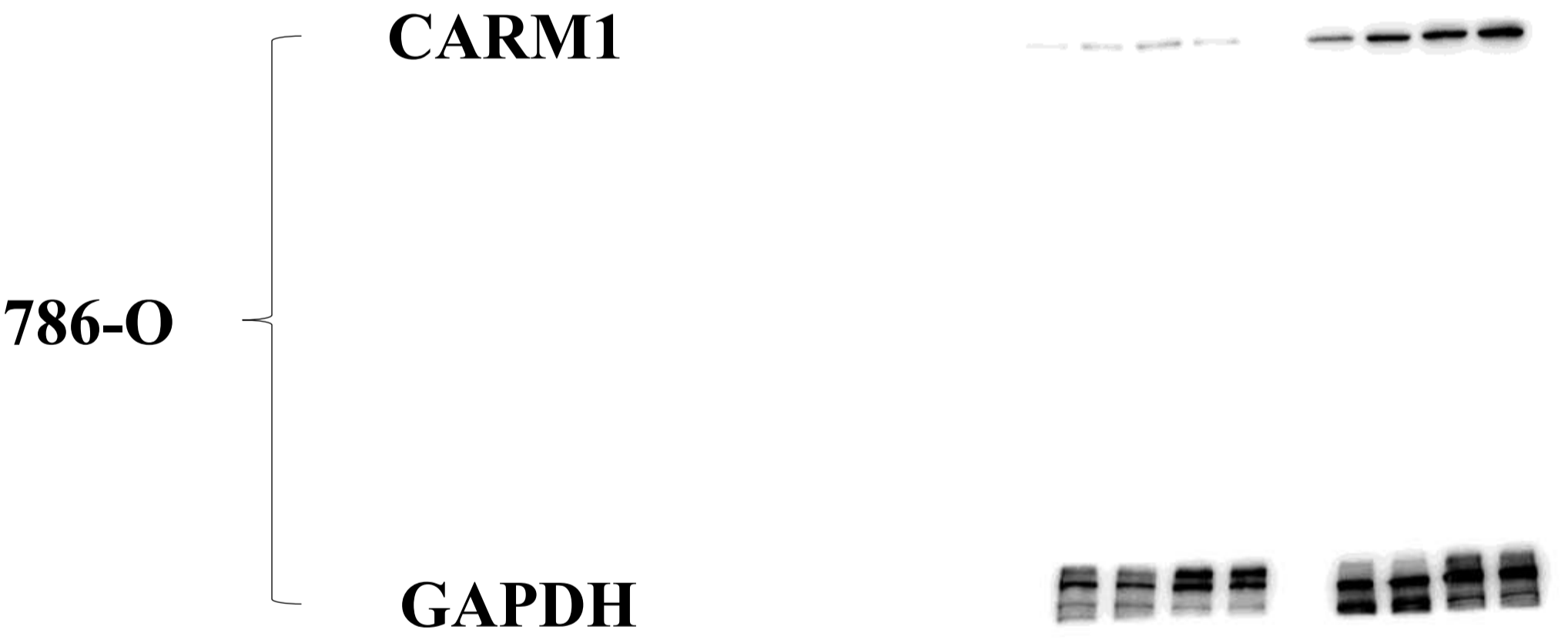

769-P

CARM1

GAPDH

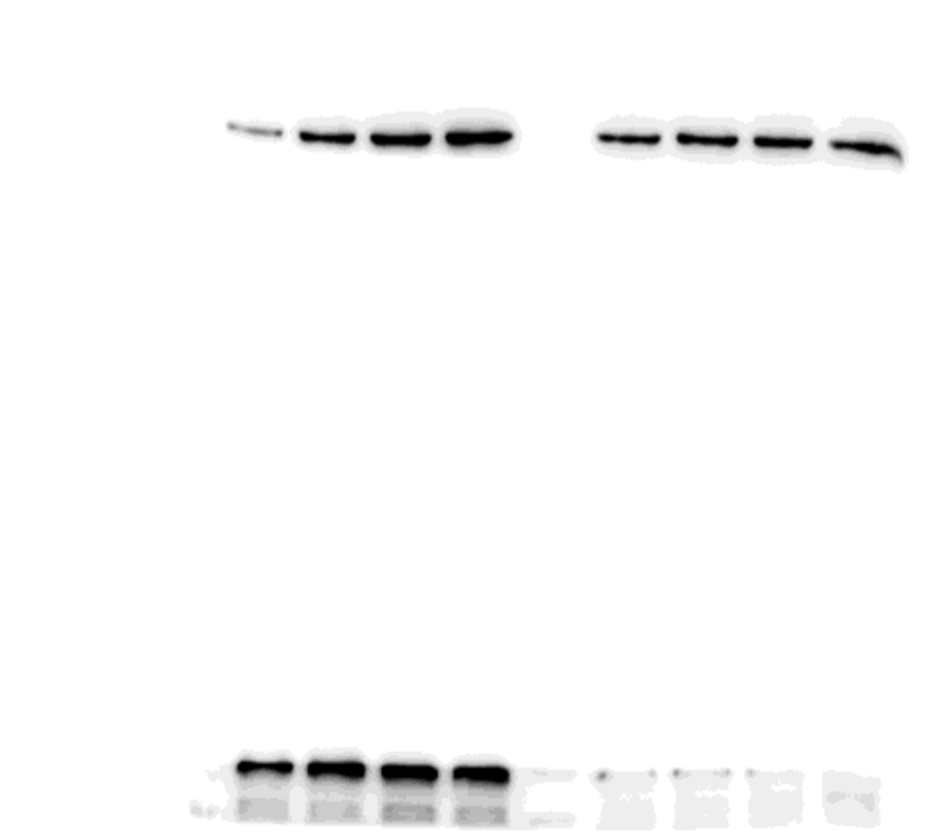

Figure 5M

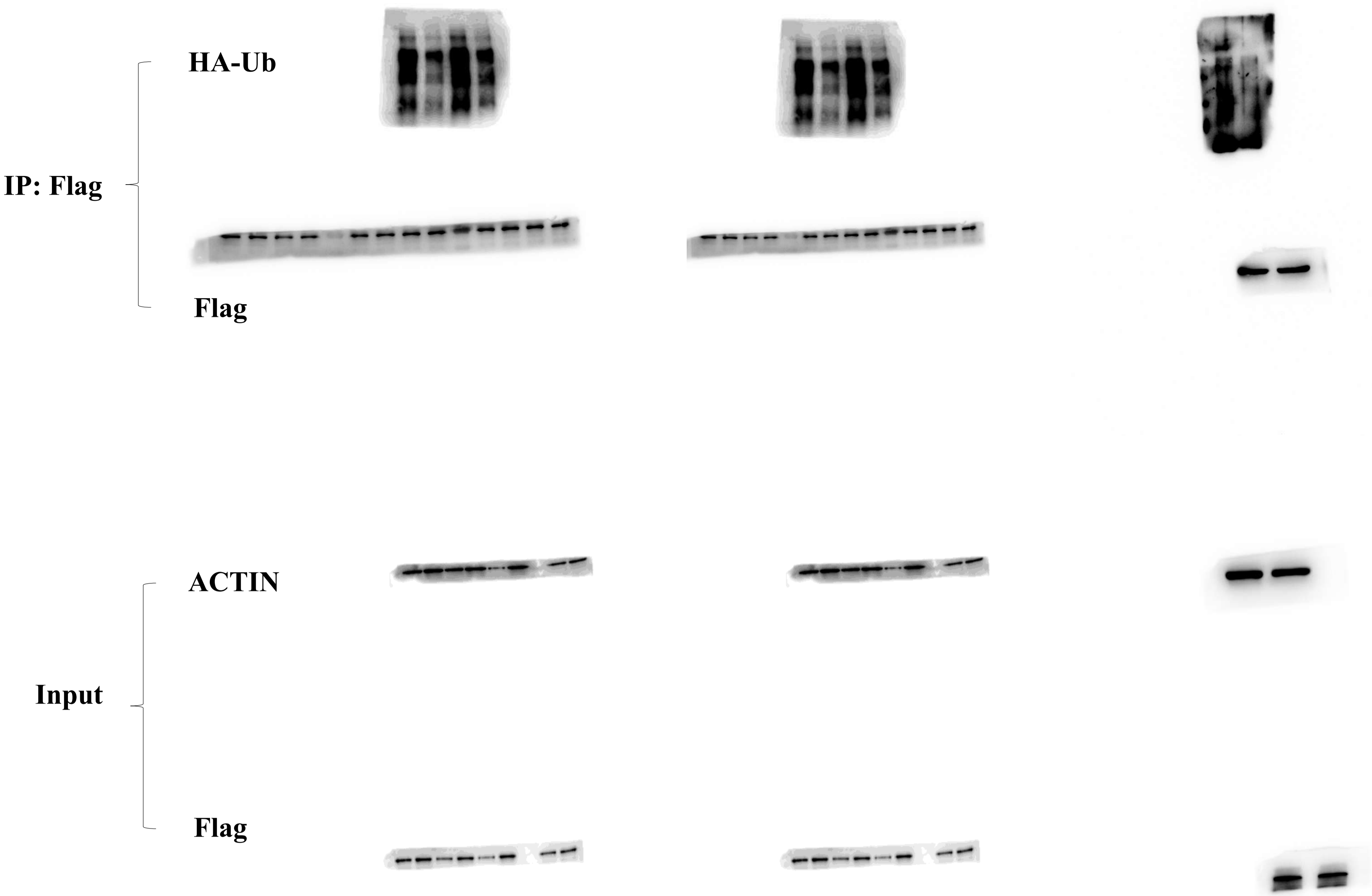

Figure 5N

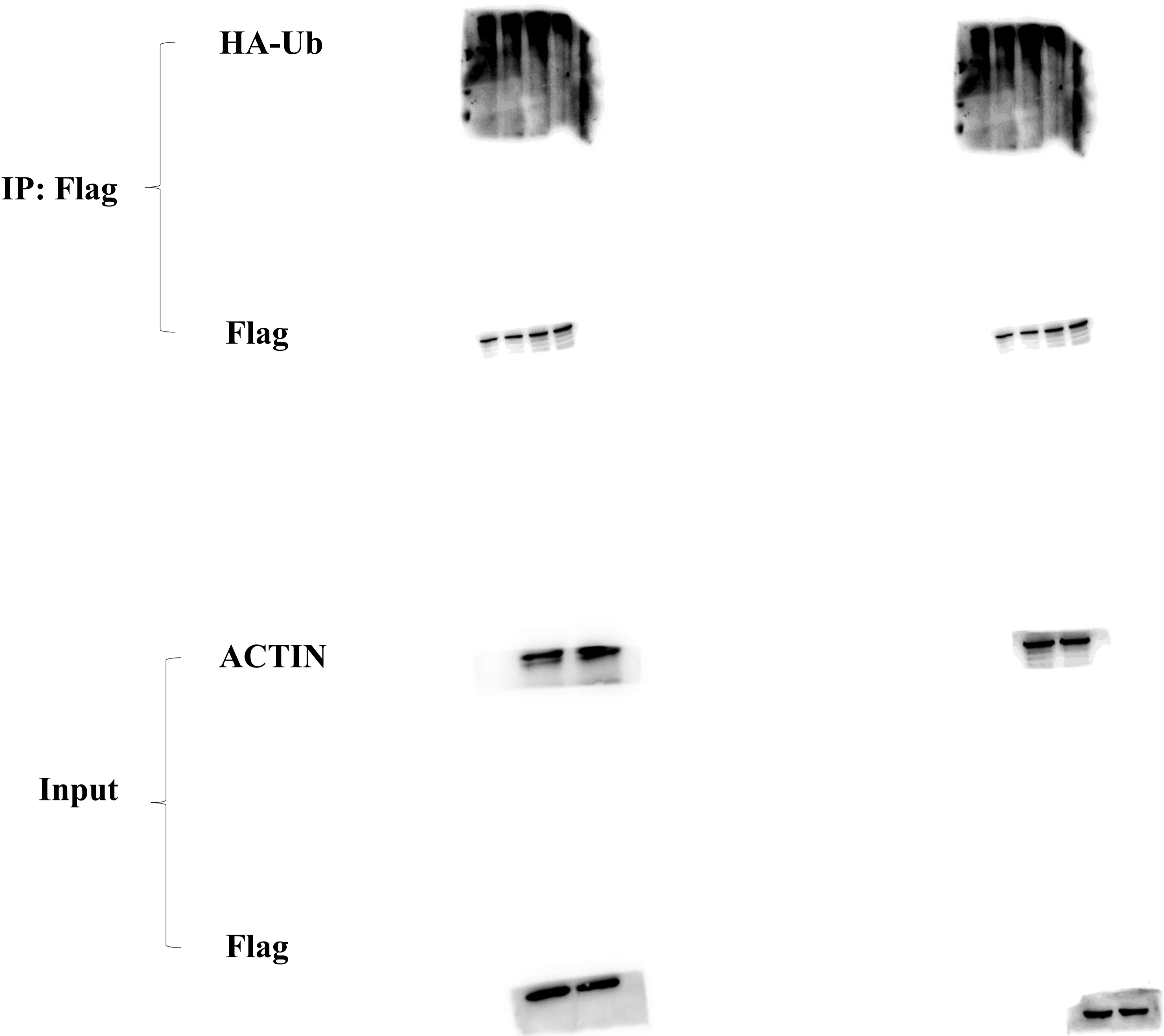

Supplement: Supplementary file 2 — uncropped Gels and Blots images [file 41419_2026_8661_MOESM2_ESM.pdf]
